# An Integrated Agriculture, Atmosphere, and Hydrology Modeling System for Ecosystem Assessments

**DOI:** 10.1029/2019MS001708

**Published:** 2020-01-24

**Authors:** L. Ran, Y. Yuan, E. Cooter, V. Benson, D. Yang, J. Pleim, R. Wang, J. Williams

**Affiliations:** 1 U.S. Environmental Protection Agency, NC, USA; 2 U.S. Environmental Protection Agency, NC, USA; 3 Benson Consulting, Columbia, MO, USA; 4 University of North Carolina at Chapel Hill, Chapel Hill, NC, USA; 5 Department of Land, Air, and Water Resources, University of California, Davis, CA, USA; 6 Blackland Research and Extension Center, Texas A&M University, Temple, TX, USA

## Abstract

We present a regional-scale integrated modeling system (IMS) that includes Environmental Policy Integrated Climate (EPIC), Weather Research and Forecast (WRF), Community Multiscale Air Quality (CMAQ), and Soil and Water Assessment Tool (SWAT) models. The centerpiece of the IMS is the Fertilizer Emission Scenario Tool for CMAQ (FEST-C), which includes a Java-based interface and EPIC adapted to regional applications along with built-in database and tools. The SWAT integration capability is a key enhanced feature in the current release of FEST-C v1.4. For integrated modeling demonstration and evaluation, FEST-C EPIC is simulated over three individual years with WRF/CMAQ weather and N deposition. Simulated yearly changes in water and N budgets along with yields for two major crops (corn grain and soybean) match those inferred from intuitive physical reasoning and survey data given different-year weather conditions. Yearlong air quality simulations with an improved bidirectional ammonia flux modeling approach directly using EPIC-simulated soil properties including NH_3_ content helps reduce biases of simulated gas-phase NH_3_ and NH_4_^+^ wet deposition over the growing season. Integrated hydrology and water quality simulations applied to the Mississippi River Basin show that estimated monthly streamflow and dissolved N near the outlet to the Gulf of Mexico display similar seasonal patterns as observed. Limitations and issues in different parts of the integrated multimedia simulations are identified and discussed to target areas for future improvements.

**Plain Language Summary:**

Computer modeling tools with land-water-air processes are important for understanding nutrient cycling and its negative impacts on air and water quality. We have developed an integrated modeling system that includes agriculture, atmosphere, and hydrology components. The centerpiece of the system is a computer system that includes an agricultural ecosystem model and tools used to connect different modeling components. The agricultural system can conduct simulations for 42 types of grassland and cropland with the influence of site, soil, and management information along with weather and nitrogen deposition from the atmosphere component. An air quality computer model then uses information from the agricultural model, such as how much ammonia is in the soil, to predict how much ammonia gets in the air. Then, the watershed hydrology and water quality model uses the information from the agricultural and atmospheric models to understand the influence of agriculture and atmosphere on water quality. The paper demonstrates and evaluates the integrated modeling system on issues mainly related to N cycling. The system performs reasonably well in comparison with survey and observation data given the configured modeling constraints. The paper also identifies and discusses the advantages and limitations in each part of the system for future applications and improvements.

## 1. Introduction

The widespread use of synthetic nitrogen (N) fertilizer along with mined phosphorus (P) has dramatically increased global agricultural land productivity since the middle of the last century (Erisman et al., 2008). More than half of the world population is now relying on the food grown with synthetic N. With the projected increase of the world population toward 10 billion by 2100 (Gerland et al., 2014), the Earth with limited agricultural land is facing unprecedented challenges to the growing demand for food and environmental protection under a changing climate (Cordell et al., 2009; Crist et al., 2017; Stewart & Lal, 2017). With scientific advancement in past decades, we have improved our understanding of the altered N cycle and its consequences for the environment and human health (Davidson et al., 2011; Fowler et al., 2013; Galloway et al., 2013). For example, excess nutrient inputs to aquatic ecosystems cause widespread eutrophication, hypoxia, and groundwater pollution (Almasri & Kaluarachchi, 2004; Goolsby et al., 2001; Smith et al., 1999). Enhanced soil nitrous oxide (N_2_O), nitric oxide (NO), and ammonia (NH_3_) emissions from fertilization (Behera et al., 2013; Stehfest & Bouwman, 2006) contribute to global warming, tropospheric ozone production, stratospheric ozone destruction, NH_4_^+^ aerosol formation, and N deposition (Aneja et al., 2001; Mathur & Dennis, 2003; Ravishankara et al., 2009). N transported and deposited in remote and sensitive ecosystems changes soil and water chemistry and biodiversity (Boyle, 2017; Stevens et al., 2004). While degraded water and air pose direct health risk to humans (Kampa & Castanas, 2008; Knobeloch et al., 2000; Lelieveld et al., 2015), rising temperature and ozone level can cause severe damages to major commodity crop production (Schlenker & Roberts, 2009; Van Dingenen et al., 2009) affecting food security.

Despite scientific progress in understanding altered N cycling and negative impacts of excess N, challenges remain, particularly regarding N sources and losses spatially and temporally over agricultural fields (Sobota et al., 2013). An integrated approach is required in assessing many aspects of the interdisciplinary science and policies on agricultural production and its interactions with ecosystems, atmosphere, and climate (Galloway et al., 2008; Greaver et al., 2016). As NH_3_ is the N-containing nutrient preferred for plant growth, NH_3_ form fertilization is dominant in agricultural production (Cao et al., 2018; Nishina et al., 2017). Given the volatility of NH_3_, it is also the major source of N in fertilized fields emitted into atmosphere. With the decrease of NO_x_ emissions in the United States (Davidson et al., 2011), the reduced-form portion of N in atmosphere from NH_3_ emissions is increasing. Agricultural practices contribute more than 80% of the global NH_3_ emissions with the majority from animal husbandry (Behera et al., 2013). NH_3_ emissions from agriculture are projected to increase continuously in the coming decades, particularly in the developing countries. In United States, 54% of total NH_3_ emissions come from animal husbandry and 30% from fertilization on agricultural cropping (Xing et al., 2013) based on the Environmental Protection Agency (EPA) 2002 National Emissions Inventory (NEI).

Accurate NH_3_ emission rates with spatial and temporal information are crucial for air quality modeling but often difficult to estimate particularly from agricultural land because the rate and timing vary with crop, region, soil, and weather conditions. Thus, NH_3_ emissions from agricultural land are often estimated using a top-down approach based on fertilizer sales information with simple seasonal variations and constant emission factors within prescribed limits. For example, NH_3_ emissions at the county level from agricultural land production in the 2011 United States (U.S.) EPA NEI (U.S. EPA, 2015) are estimated using the Carnegie Mellon University (CMU) Ammonia Model v.3.6 (Goebes et al., 2003) with county-level fertilizer consumption data or estimates. Fertilizer application timing is based on constant state-level climatological averages, and fertilizer applications are distributed monthly to agriculture land areas. Thus, this top-down approach does not account for variations in rates and timing of fertilizer applications by production types and geographical areas with different soil and micrometeorology conditions.

A cropping system, which has complete soil and plant carbon and nutrient cycles along with hydrology and management practices including fertilization, is required to more accurately estimate spatial and temporal soil NH_3_ which is available for volatilization. Given the bidirectional nature of ammonia fluxes (upward or downward), it is important to include bidirectional processes in estimating NH_3_ emissions such as resistance approaches described by Sutton et al. (1998). Combining with atmospheric conditions, resistance approaches use NH_3_ compensation points, which were first introduced by Farquhar et al. (1980) for flux computation (Massad et al., 2010). To estimate the bidirectional NH_3_ flux from managed agricultural soils, Cooter et al. (2010) tested a resistance and compensation point flux approach (Nemitz et al., 2001) integrating the deposition model (Pleim & Ran, 2011) in the Community Multiscale Air Quality (CMAQ, https://www.epa.gov/cmaq) with components of the Environmental Policy Integrated Climate (EPIC, https://epicapex.tamu.edu/epic/) model. EPIC is a sophisticated agricultural ecosystem model (Williams, 1995; Williams et al., 1984) developed with the support from the U.S. Department of Agriculture (USDA), and CMAQ is a numerical air quality model (Appel et al., 2017; Byun & Schere, 2006) developed at the U. S. EPA. Based on their integrated results with good daily and accumulated monthly NH_3_ estimates, U.S. EPA developed the Fertilizer Emission Scenario Tool for CMAQ (FEST-C, https://www.cmascenter.org/fest-c/) system to facilitate the generation of agricultural soil properties with N fertilization information for bidirectional NH_3_ flux modeling (Pleim et al., 2013) in CMAQ. The system was first designed to integrate EPIC with a mesoscale meteorology and air quality modeling system, which includes Weather Research and Forecast model (WRF, http://www2.mmm.ucar.edu/wrf/users/; Skamarock et al., 2008) and CMAQ.

The released FEST-C system contains a Java-based interface and EPIC adapted to regional applications along with built-in database and connection tools. Since the first release of FEST-C v1.0 in 2013 (Ran et al., 2011), the system has gone through many updates and enhancements up to the recent release of FEST-C V1.4 (Ran et al., 2018). Cooter et al. (2012) presented the system with detailed descriptions and evaluations of simulated EPIC and CMAQ. They showed that using EPIC fertilization in CMAQ with a bidirectional NH_3_ flux model results in more spatially and temporally resolved estimates of NH_3_ emissions from the agricultural land and in improved estimation of ambient particulate nitrate concentrations. Since then, the results from FEST-C have been used in many research and assessment studies, such as the integrated ecosystem sustainability assessment of increased corn production in the United States under corn cellulose biofuel scenarios by Cooter et al. (2017) and health impact assessment of ground water contamination from the increased corn production by Garcia et al. (2017). In FEST-C v1.4, the system is enhanced to better integrate with the Soil and Water Assessment Tool (SWAT, https://swat.tamu.edu/) modeling system (Arnold et al., 1998 & 2012) to improve our understanding of agricultural production, weather, and N deposition impacts on hydrology and water quality in the Mississippi River basin and on hypoxia in the Gulf of Mexico (GOM; Yuan et al., 2018). These enhancements have advanced the system capabilities from simply supporting CMAQ simulations, to becoming a valuable tool for integrated ecosystem assessments of air, land, and water quality considering social drivers and human and ecological outcomes as described by Cooter et al. (2013). Therefore, it is important to thoroughly describe and evaluate the integrated system in order to understand the strengths and limitations for future improvements and applications.

The goal of this paper is to present the current release of FEST-C v1.4 and to demonstrate and evaluate integrated modeling of agriculture, atmosphere, and hydrology and water quality mainly focusing on issues related to N cycling. The FEST-C system is presented in section 2 with an emphasis on integrated components, recent updates, and input of agricultural land and N deposition data. Integrated modeling configuration, results, and evaluation are demonstrated in section 3 with a focus on overall performance of N budget from agricultural simulations. Simulated results are evaluated in detail against survey and measurement data, and added values and limitations of this integrated system are identified and discussed. Conclusions and future work are presented in the last section.

## 2. FEST-C

The Java-based interface FEST-C system is the central platform that guides users through EPIC simulations for any CMAQ grid domain over the conterminous United States (CONUS) and facilitates integrated modeling of agriculture, atmosphere, and hydrology (Ran, Cooter, Yang, Benson, et al., 2018). Integration among the models is offline and through input-output connection tools. The system uses open-source software with public-available standalone tools and models, which are being developed and applied continuously by different organizations. Simplicity and easy upgrades and updates are the principles guiding the design and development of the system in order to minimize maintenance efforts in light of constantly changing computer technology and software tools. The system along with its required components (e.g., Spatial Allocator and VERDI) has been developed for Linux system applications and released at the Community Modeling and Analysis System (CMAS) Center (https://www.cmascenter.org/) to the public. To apply this system to other regions, users have to modify the databases (particularly agricultural land, site information, and soil data input) that are created for FEST-C and to change the agricultural management file generation, which is associated with regional management and fertilization representation. For example, Fu et al. (2015) demonstrated the feasibility in adapting this system for CMAQ air quality simulations in China with bidirectional NH_3_ flux modeling.

### 2.1. Integrated Models

**Figure 1.**
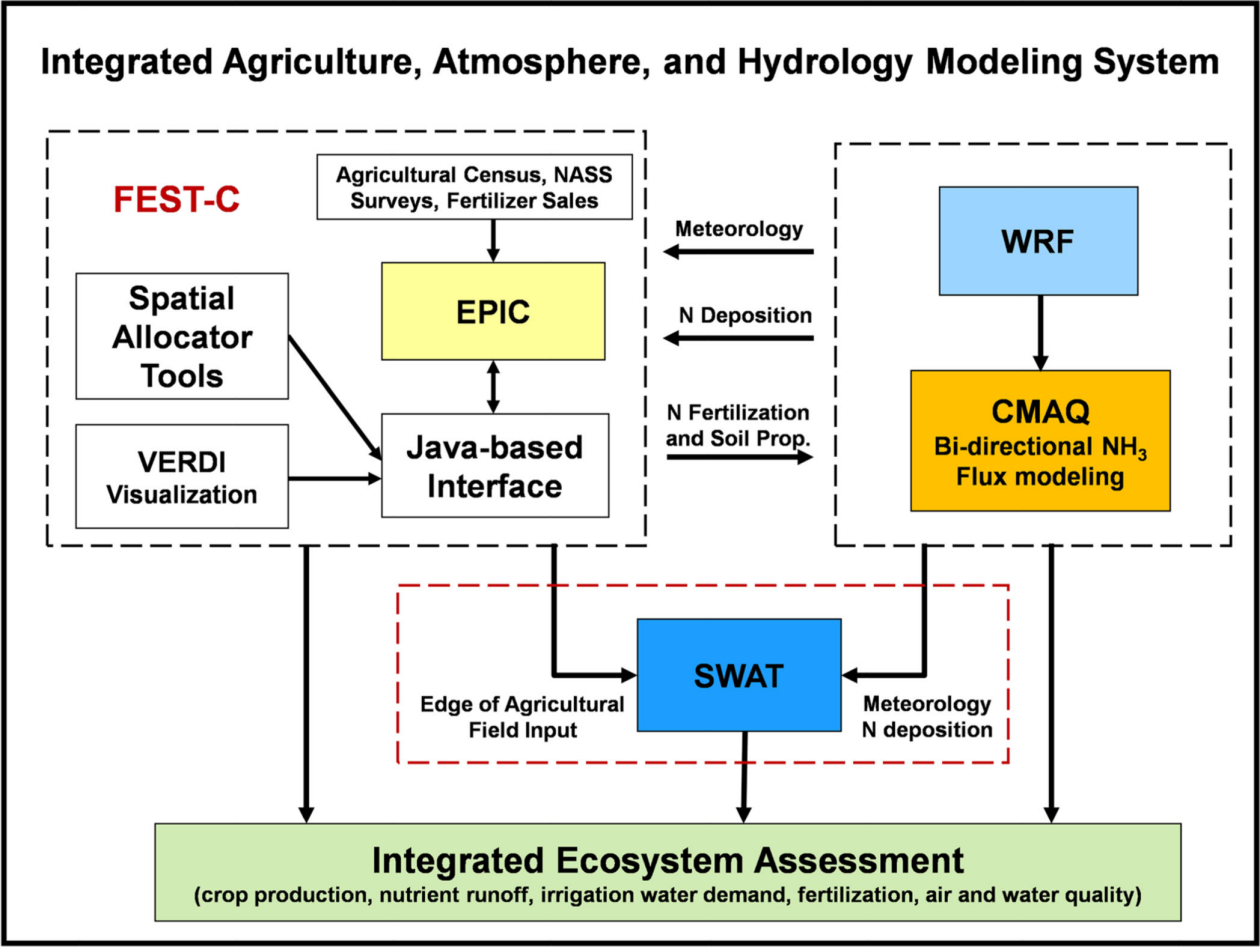
Fertilizer Emission Scenario Tool for CMAQ (FEST-C) V1.4 with integrated Environmental Policy Integrated Climate-Weather Research and Forecast-Community Multiscale Air Quality-Soil and Water Assessment Tool (EPIC-WRF-CMAQ-SWAT) modeling system. FEST-C with EPIC is displayed in the left dash box. The major enhancement of this version is the SWAT integration which is displayed in the red dash box.

The process diagram of FEST-C including EPIC and tools (left dash box) and linkages among models are displayed in Figure 1. EPIC is a cropping model, which has long been used in a range of applications related to field-scale soil erosion, crop productivity, irrigation, climate change, and water quality around the world (e.g., Benson et al., 1989; Elliott et al., 2015; Rosenzweig et al., 2014; Wriedt et al., 2009). In this system EPIC simulations can be conducted for CMAQ grid domains at different resolutions and in any of the four WRF projection coordinate systems: longitude/latitude, Lambert Conformal Conic, Universal Polar Stereographic, and Mercator. The regional management representation, soil data, and weather input required are all described in detail by Cooter et al. (2012). The fertilizer use by crop, type, timing, and application method (not amount) and tillage from fertilizer sales and survey information, which are reasonably representative of the ten U.S. agricultural production regions, are incorporated into the management files. Fertilization amount is simulated dynamically based on the N demand by crop, growth stage, and region along with different stress mechanisms such as by heat and water. The realism of the management representation adds measured information to the physiological cycling of nutrients, hydrologic modeling of movement, and impacts on air and water quality. Meanwhile, with the N demand-based fertilization approach the system simulates crop growth dynamically with links to external N sources (fertilization, N deposition, and fixation) and internal source of N from soil organic matter (SOM) as well as weather conditions. The soil input profile with carbon and organic matter is generated using the Baumer soil data files processed from the USDA Natural Resources Conservation Service (NRCS) soil databases (Baumer, 1992). For stabilizing the nutrient pool to the prescribed management, the system is first initialized in the spinup with100 years for potatoes and 25 years for all other types based on system performance. Averaged last 5-year spinup results are used as initial soil and to guide fertilization for a specific weather-year EPIC simulation that uses WRF/CMAQ weather and N deposition.

As a combined meteorology and air quality modeling system, WRF/CMAQ is an important decision support tool that is widely used for increasing our understanding of the chemical and physical processes contributing to air quality impairment and for facilitating the development of policies to mitigate harmful effects of air pollution on human health and the environment (e.g., Cohan et al., 2007; Compton et al., 2011; Wang et al., 2016). The WRF/CMAQ system provides daily average weather input and N deposition to FEST-C EPIC for simulating plant growth with planting/harvesting, fertilization, production, hydrology, and complete soil biogeochemical properties under various management practices and soil conditions. In return, FEST-C extracts EPIC-simulated daily N fertilization information and soil properties with pH, soil moisture, and NH_3_ conditions, which are required input for CMAQ bidirectional NH_3_ modeling.

SWAT is a powerful tool, which has long been used to assess the impact of weather/climate, soil, and land management practices on water, sediment, and agricultural chemical yields at the watershed scale (Abbaspour et al., 2015; Saleh et al., 2000; White et al., 2014). Since both EPIC and SWAT are developed at Texas A&M University (TAMU) with the support from USDA, the two systems contain similar modules for some physical processes. For example, SWAT contains a simplified version of EPIC cropping component for modeling agricultural land. Thus, SWAT linked with FEST-C EPIC not only has the agricultural land fully-simulated by EPIC but also maintains some consistency in biogeochemical processes, which is important in integrated ecosystem assessments. Water and nutrient runoff at edge of agricultural fields from EPIC as well as daily average weather and N deposition information from WRF/CMAQ can be extracted to each watershed for SWAT simulations. Thus, the simple interface facilitates integrated ecosystem assessments on agricultural production, nutrient cycling, and land-water-air quality under different managements.

### 2.2. New Updates

In addition to the enhanced interface and tools for SWAT integration (displayed by the red dash box in Figure 1), the released system has many updates to FEST-C EPIC, which was originally based on EPIC version 0509. Some parameterizations related to the carbon and N cycles are being further tested and updated following the advancement of the model (e.g., Izaurralde et al., 2017). The EPIC is also updated with an additional approach in computing percolation and lateral subsurface flow following a more recent version of the model (Doro et al., 2017). The improved N cycling (particularly for denitrification) and hydrology processes help reduce high biases of N fertilization and runoff from the previous version. Meanwhile, the depth of the drain tile (set to 750 mm) is added to the soil input based on the soil hydrologic group. Thus, the FEST-C EPIC has an option for users to include the tile drainage process. Meanwhile, 2012 county crop type census information and two N deposition data sets over the CMAQ 12-km CONUS domain are added to the system for land use and N deposition selection in EPIC simulations. As agricultural land and N deposition inputs are important and unique in this modeling system over the CONUS, they are described in detail below.

### 2.3. Agricultural Land and N Deposition Input

#### 2.3.1. Agricultural Land

**Figure 2.**
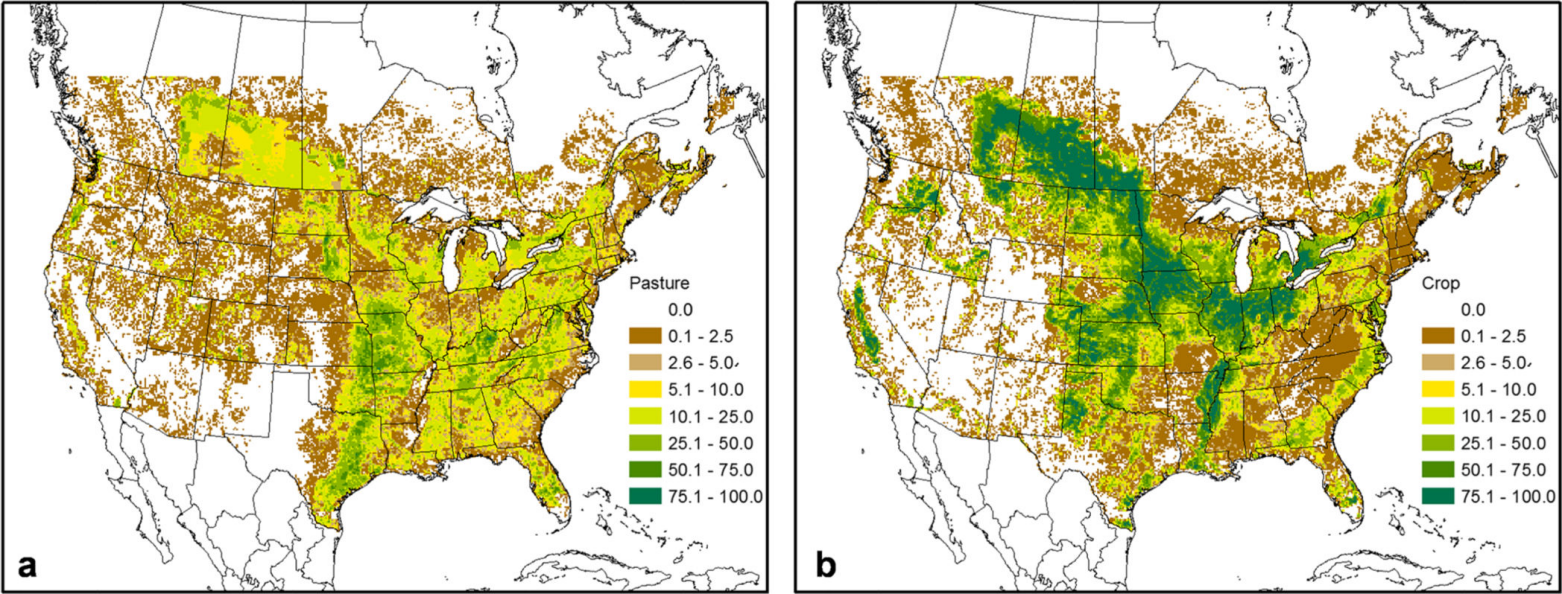
(a) Managed grassland or pasture and (b) cropland percent in the Community Multiscale Air Quality (CMAQ) 12-km domain grid cells computed from 2011 National Land Cover Database/Moderate Resolution Imaging Spectroradiometer (NLCD/MODIS), 2012 U.S. Department of Agriculture (USDA) National Agricultural Statistics Service (NASS) Census, and 2011 Canada Census of Agriculture data in Fertilizer Emission Scenario Tool for CMAQ (FEST-C).

FEST-C EPIC simulates 21 different agricultural production systems ranging from managed grassland (e.g., hay and alfalfa) to cropland (e.g., corn grain and soybean) differentiated by rainfed and irrigated categories, which are listed in Table S1 in the supporting information as 42 production types. As land use data with consistent agricultural land information are crucial in this integrated system, the linked models all use the National Land Cover Database (NLCD) for CONUS. FEST-C EPIC and WRF/CMAQ use the land use processing tools developed in Spatial Allocator (SA, https://www.cmascenter.org/sa-tools/) for the CMAQ air quality community (Ran et al., 2015) by processing directly downloaded NLCD data (Homer et al., 2015) for United States and Moderate Resolution Imaging Spectroradiometer (MODIS) data (Friedl et al., 2010) for areas outside United States. The system enables users to select any version of NLCD data sets for years of 2001, 2006, and 2011. The county-level production type fractions over the corresponding periods, generated from the USDA National Agricultural Statistics Service (NASS) Census of Agriculture (COA) data and census data from Canada, are used to partition the production type fractions at each model grid cell. Figure 2 displays the FEST-C generated total managed grassland (2a, types 1 to 6 in Table S1) and cropland (2b, types 7 to 42 in Table S1) percent in CMAQ 12-km domain grid cells from 2011 NLCD/MODIS data (Text S1).

The production type fractions are used in not only determining where EPIC simulations are conducted in the domain but also aggregating EPIC output from grid cells for regional evaluation and analysis. Thus, it is important to understand the overall accuracy of NLCD pasture/hay and cropland areas, particularly in comparison with the USDA NASS COA agricultural land data as most of EPIC results are evaluated against the COA reports. While NLCD class accuracy (Level II) is around 83% in comparison with reference samples interpreted from Google Earth™ imagery (Wickham et al., 2017), NLCD tends to have higher uncertainty in distinguishing among cropland and natural and managed grassland (Goslee, 2011; Maxwell et al., 2008). The FEST-C agricultural land computed from the three different year NLCD is compared with USDA COA data aggregated from the state-level report (Text S2 and Table S2). Total NLCD agricultural land from 2001 to 2011 shows very little change domain wide with the cropland areas very similar to the COA cropland area. However, the NLCD managed grassland may be underestimated because of the difficulty in distinguishing managed grassland from natural grassland in satellite data classification. The irrigated land for FEST-C 2011 production types agrees well with the total irrigated land reported from 2012 COA. Given the difference in agriculture land between NLCD and COA, it is important to keep some perspectives in evaluating and applying NLCD-based EPIC results.

**Figure 3.**
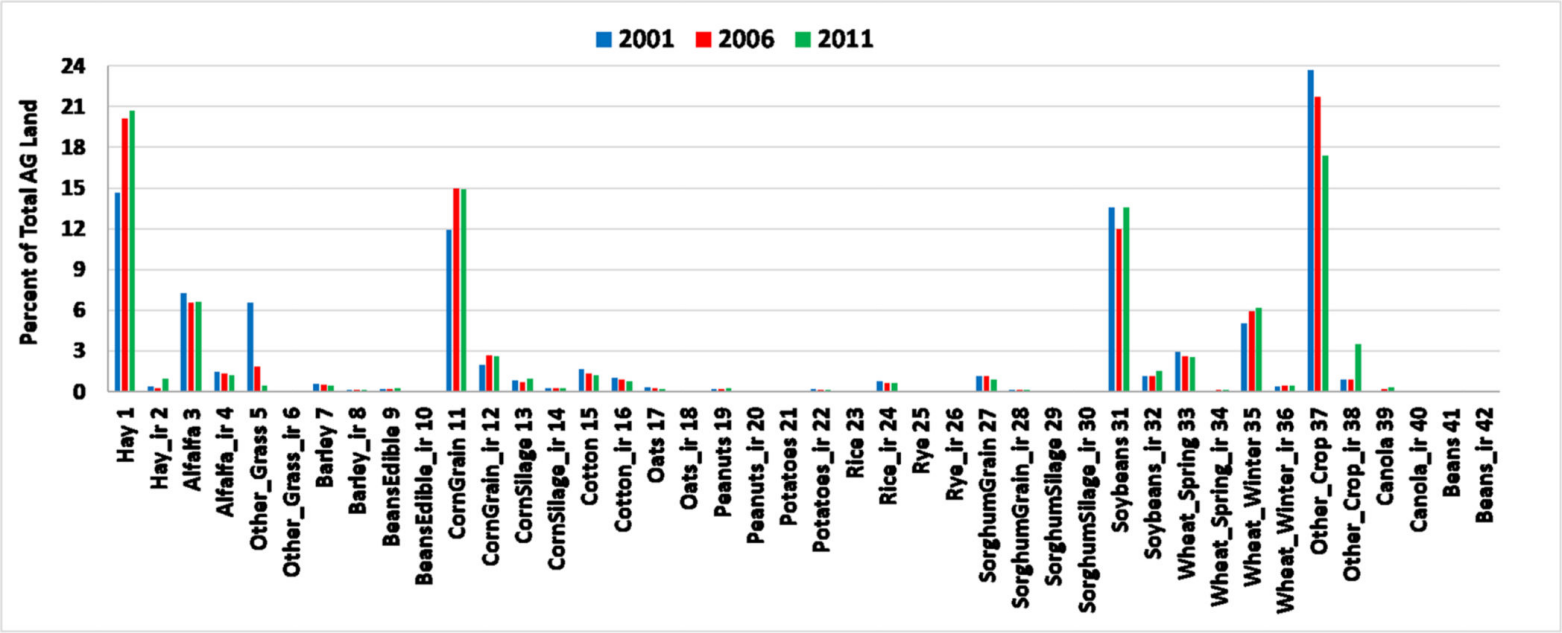
Percent of the production area to the total agricultural land for Fertilizer Emission Scenario Tool for CMAQ (FEST-C) 2001, 2006, and 2011 land use data sets over the conterminous U.S. domain. X axis—42 grassland and cropland types and names for 2001 (blue), 2006 (red), and 2011 (green). Y axis—percent to the total agricultural land over the corresponding year. Note that types 1 and 2 are for rainfed hay and irrigated hay, and types 3 and 4 for rainfed alfalfa and irrigated alfalfa. The 42 type numbers and names are also listed in Table S1.

Figure 3 shows the percent of each production type to the total agricultural land in CONUS for the three land use dataset years generated in FEST-C. The dominant production types in United States are hay (types 1 and 2), alfalfa (3 and 4), corn grain (11 and 12), soybean (31 and 32), wheat (33 to 36), and other crops (37 and 38). Although the total agricultural land area stays relatively the same over the period, the areas of different production types do change following the COA data (Text S3 and Figure S1 in the supporting information). With the change of production types over the available NLCD years, FEST-C can be used to facilitate impact assessments of production shifts on N budgets and, in turn, on regional air and water quality. In addition, the future production composition as well as land use change can be easily incorporated into the system for future scenario assessments as demonstrated by Cooter et al. (2017).

#### 2.3.2. N Deposition

Atmospheric N deposition can be a significant source that influences N cycling in ecosystems, particularly those with N limitation. EPIC allows users to specify average N concentration in rainfall in an input parameter file though dry deposition is not considered in the standard model due to the difficulty in obtaining dry deposition information. As dry deposition can be a major pathway for the removal of trace N chemical species from the atmosphere, and volatilized NH_3_ from fertilization can greatly increase N deposition in agricultural areas, the influence of spatially and temporally resolved dry and wet N deposition from CMAQ is considered for agricultural production in this integrated EPIC. Dry and wet N deposition in oxidized, reduced, and organic forms estimated from CMAQ can be selected for ingestion into the surface soil layer to influence soil N processes. The current release of FEST-C includes two 5-year average daily N deposition data sets representing the 2002 to 2006 and 2006 to 2010 periods, processed from CONUS CMAQ 12-km grid resolution simulations (Zhang et al., 2019), for EPIC N deposition selection in addition to the default option and year-specific CMAQ N deposition.

**Figure 4.**
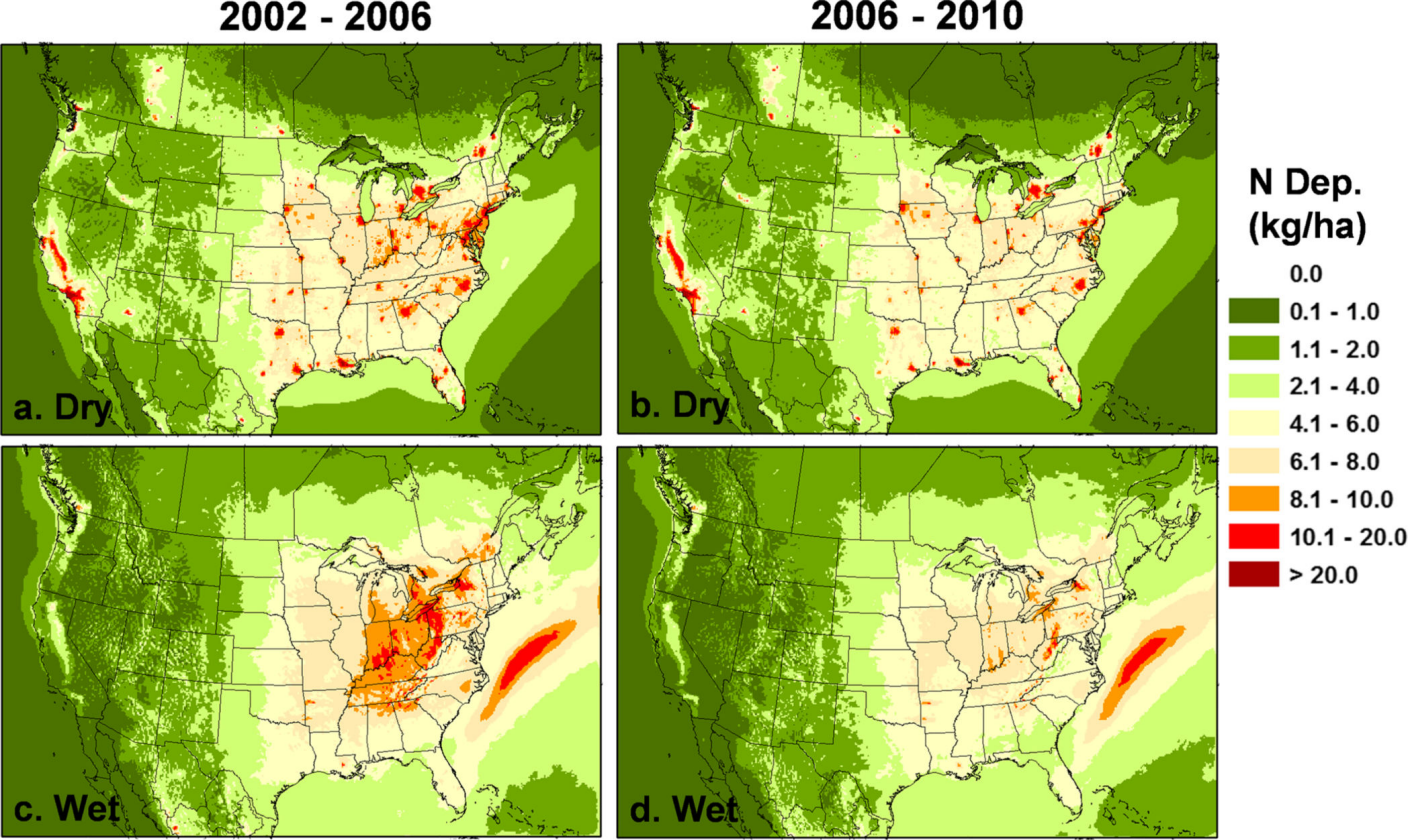
Yearly total (a and b) dry and (c and d) wet N deposition (kg ·ha^−1^ · year^−1^) from the two 5 year average CMAQ simulations (a and c for 2002 to 2006 and b and d for 2006 to 2010) over the conterminous United States (CONUS) 12-km domain cells in Fertilizer Emission Scenario Tool for CMAQ (FEST-C).

Figure 4 shows that the yearly wet and dry N deposition from the two average daily N deposition data sets and domain-wide average comparison is displayed and described in Figure S2 and Text S4. Both dry and wet N deposition shows a decreasing trend over the 2006–2010 period (Figures 4b and 4d) relative to the 2002–2006 period (Figures 4a and 4c) with dry deposition reduction in the east around major metropolitan areas and wet deposition reduction in the Ohio Valley region. The N deposition reduction reflects tightened standards by U.S. EPA on NO_X_ (NO + NO_2_) emissions from large stationary sources including power plants and from onroad vehicles before 2007 (Simon et al., 2014). With the control of NO_x_ emissions, the reduced-N deposition is becoming more important to the total N deposition budget as the oxidized-N deposition declines over United States (Li et al., 2016; Zhang et al., 2018). With CMAQ N deposition input options along with WRF meteorology to both EPIC and SWAT, the impacts of NO_X_ emission controls on agriculture and hydrology can be consistently explored in this integrated system.

## 3. Integrated Modeling and Evaluation

Integrated modeling is conducted for the system demonstration and evaluation. Table 1 summarizes the simulations conducted for agriculture EPIC, air quality CMAQ, and hydrology and water quality SWAT with integrated input. The EPIC simulations are conducted and evaluated for each of three consecutive years—2010, 2011, and 2012 over the CONUS. Simulated meteorology and N deposition from WRF version 3.4 and CMAQ version 5.0 over the CONUS domain for the years are used to drive EPIC application simulations. The retrospective WRF/CMAQ simulations and estimated N deposition are described and evaluated in detail by Zhang et al. (2019). As EPIC is the centerpiece of this integrated modeling, the evaluation includes important aspects of agricultural production such as water budget, yield, and N budget, which is the key connecting to air and water quality and is the focus of the evaluation.

Integrated atmosphere and hydrology modeling is demonstrated with CMAQ simulations over the CONUS 12-km grid domain for the year of 2011 and SWAT simulations over the Mississippi River Basin (MRB) for the period from 2010 to 2012. Soil physical and chemical properties along with fertilization information for each CMAQ grid cell for the 42 production types are extracted from the 2011 EPIC simulation described above into CMAQ-ready domain-wide daily NetCDF format files through the FEST-C interface (Ran, Cooter, Yang, Benson, et al., 2018). SWAT-ready edge of field input files for the eight-digit hydrologic unit code (HUC) watersheds in MRB are also generated in FEST-C by extracting daily nutrient, water, and sediment runoffs from the EPIC simulation. Weather (radiation, average temperature, precipitation, relative humidity, and wind speed) and N deposition (reduced/organic and oxidized pools) information are from processed daily average WRF/CMAQ output. Simulated ambient gas-phase NH_3_ concentration and NH_4_^+^ wet deposition from CMAQ and stream flow and dissolved N content load from SWAT are evaluated against observations.

**Figure 5.**
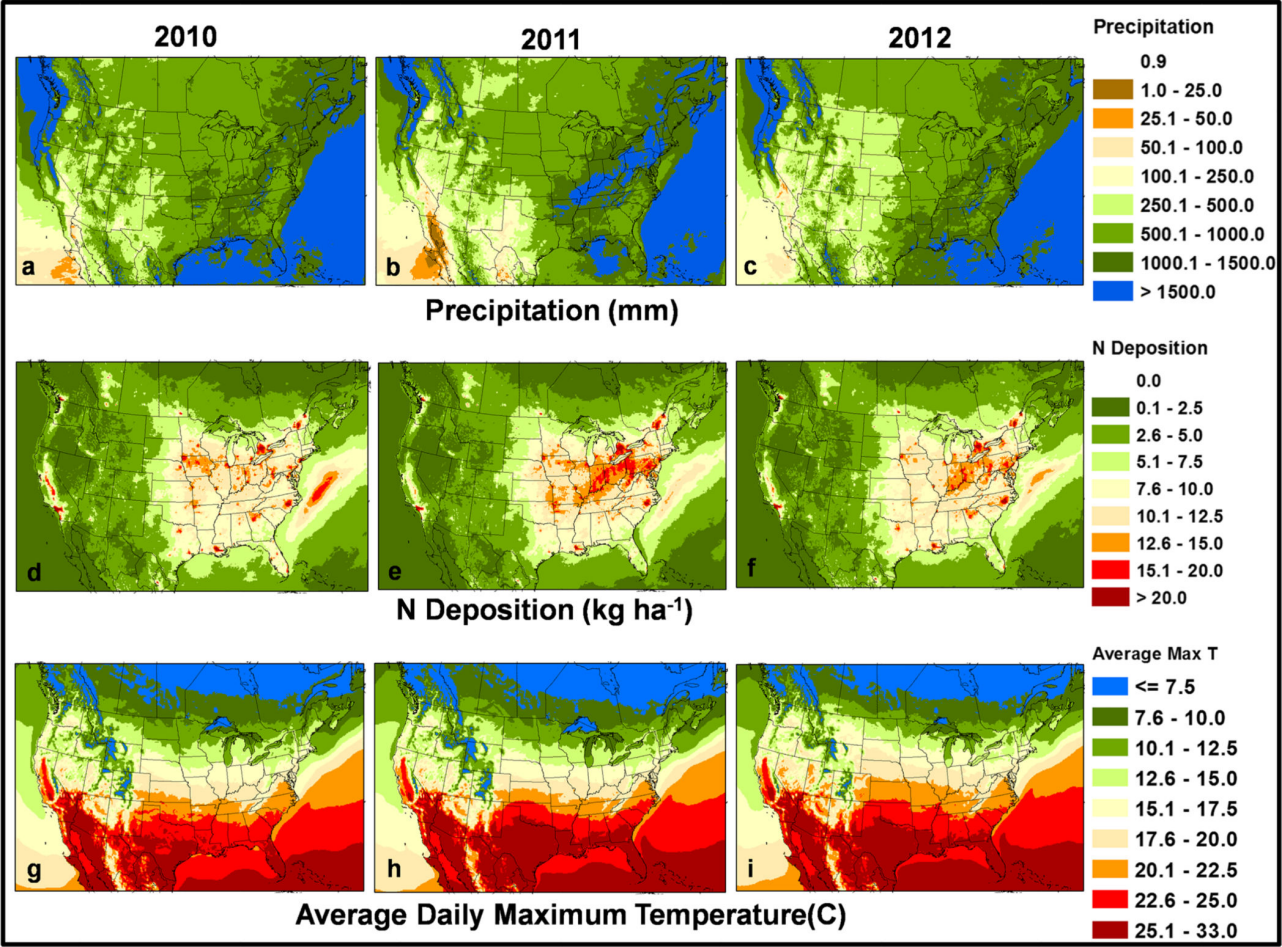
(a–c) Annual total precipitation and (d–f) N deposition and (g–i) average daily maximum temperature on the 12-km grid domain from Weather Research and Forecast/Community Multiscale Air Quality (WRF/CMAQ) simulations over 2010, 2011, and 2012.

The precipitation, N deposition, and daily maximum temperature, which are key inputs to EPIC and SWAT, are demonstrated in Figure 5 for the 3 years. The 2010 precipitation (Figure 5a) and temperature (Figure 5g) are relatively normal in historical perspective. For 2011, despite high precipitation in the Ohio Valley and Northeast, the CONUS as a whole is drier than average (Figure 5b) and particularly Texas suffered the worst drought in recent decades (Nielsen-Gammon, 2012). Starting from late 2011 California experienced the worst multiyear drought in the recent century (Griffin & Anchukaitis, 2014). Year 2012 is the driest year for the CONUS with lower precipitation from the Intermountain West, through the Great Plains and into the Midwest where agricultural land is dominant (Figure 5c). Meanwhile, the higher average daily maximum temperature moved further north (Figure 5i). Over the 3 years, N deposition shows relatively similar patterns low in the west and high from the central to the east (Figures 5d, 5e, and 5f). Major metropolitan areas and some intensive agricultural regions such as the Central Valley, the Corn Belt states, and eastern North Carolina tend to have high deposition due to high NO_x_ and NH_3_ emissions. The Central Valley in 2010 and the Ohio Valley and Northeast in 2011 show the highest deposition due to much more wet deposition from higher precipitation.

### 3.1. Integrated Agriculture

EPIC is configured with the Hargreaves method for daily evapotranspiration, variable daily curve number with depth soil water weighting for runoff estimation, curve number estimate for infiltration, modified Universal Soil Loss (MUSL) equation for water erosion, Armen Kemanian method for denitrification, and 4-mm slug flow method for percolation and subsurface flow computation (Doro et al., 2017; Williams, 1995). Automatic fertilization and irrigation are triggered by computed plant N and water stress factors. For simplicity, all irrigation is assumed to use the sprinkler system. The tile drainage process is included in the agricultural land hydrology, and the atmospheric CO_2_ level is set to be 392 ppm, close to the global average in earlier 2010s. The 2011 agricultural land fractions are used in simulations for all 3 years. The system is first initialized with the 5-year average CMAQ N deposition data over the 2006–2010 period in the spinup. The simulation uses the same site, land use, management, and soil information from the same spinup with WRF/CMAQ input for each of the years. Thus, the EPIC difference purely reflects sensitivities of agricultural land simulations to the spatial and temporal changes of atmospheric conditions over the period.

#### 3.1.1. Water Budget With Irrigation Water Demand

**Figure 6.**
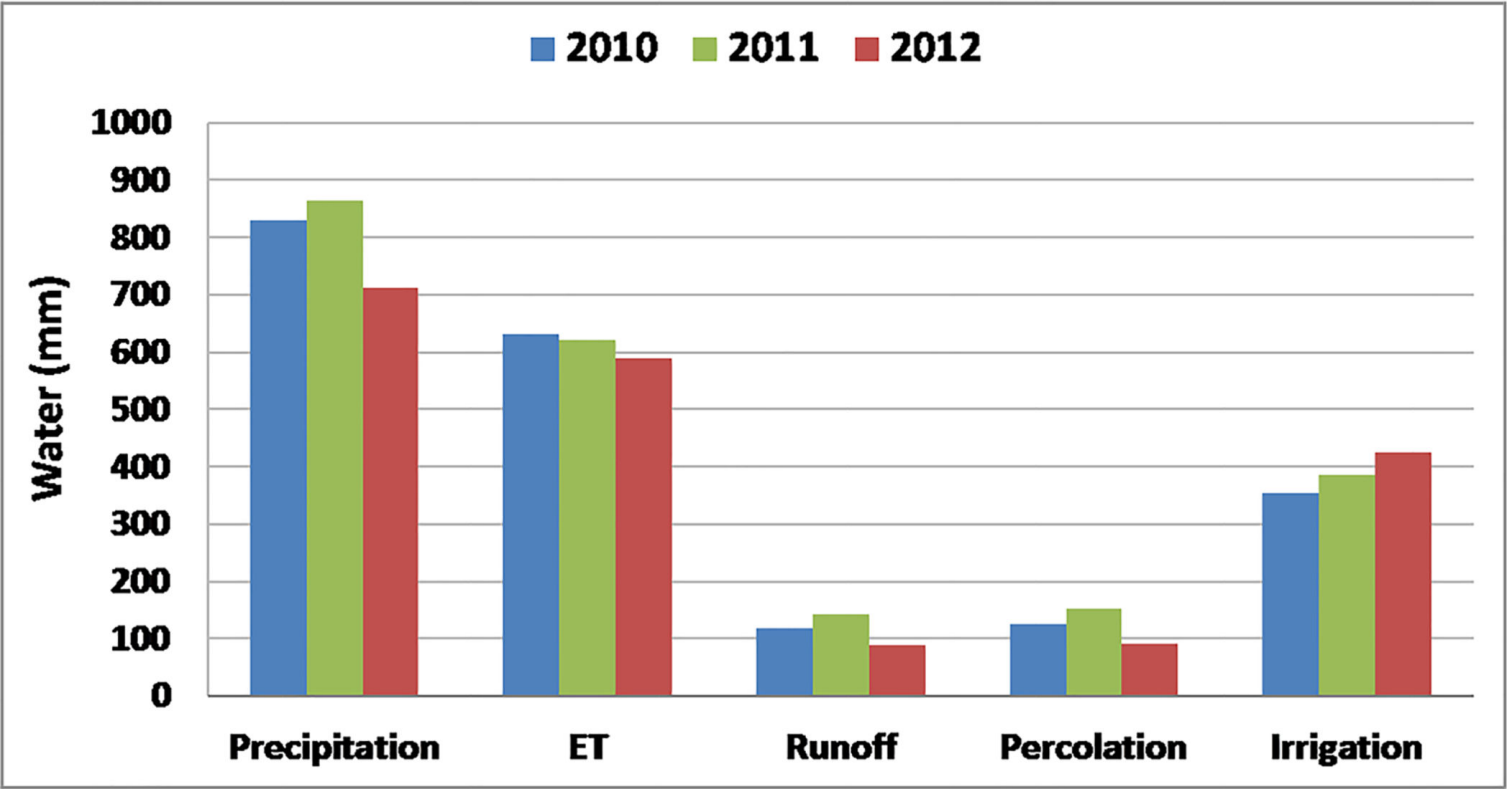
Annual water budget from Environmental Policy Integrated Climate (EPIC) simulations over the conterminous United States (CONUS). Area-weighted average precipitation, evapotranspiration (ET), runoff (surface and subsurface including tile drainage), and percolation are for all agricultural production areas (177,588,407.5 ha). Area-weighted average irrigation demand is for the irrigated areas (22,788,210.8 ha). Note that return flow is not simulated in this version of the system.

The domain-wide water budget is displayed in Figure 6 for demonstrating the sensitivity of hydrology and irrigation water demand to the changes in weather conditions over the 3 years. Evapotranspiration (ET) shows a decreasing trend from 2010 to 2012, while the demand for irrigation increases due to the worsening dry conditions as discussed above. Even though the highest domain-wide precipitation input (865 mm averaged on the agricultural land) is from 2011, most of the increase comes from high precipitation in the east (Figure 5) where the climate is wetter and water is not usually the limiting factor for production. Thus, the excessive precipitation results in high runoff (includes surface and subsurface with tile drainage) and percolation on the average for 2011. The significant reduction of precipitation by more than 100 mm in the driest year of 2012 results in the lowest ET falling below 600 mm, reduced runoff and percolation down to around 90 mm, and much higher irrigation reaching 425 mm on irrigated land. The runoff and percolation have similar magnitude in the system and are small in comparison with the others. However, nutrients in those relatively small partitions are a major concern in water quality for pollution reduction.

**Figure 7.**
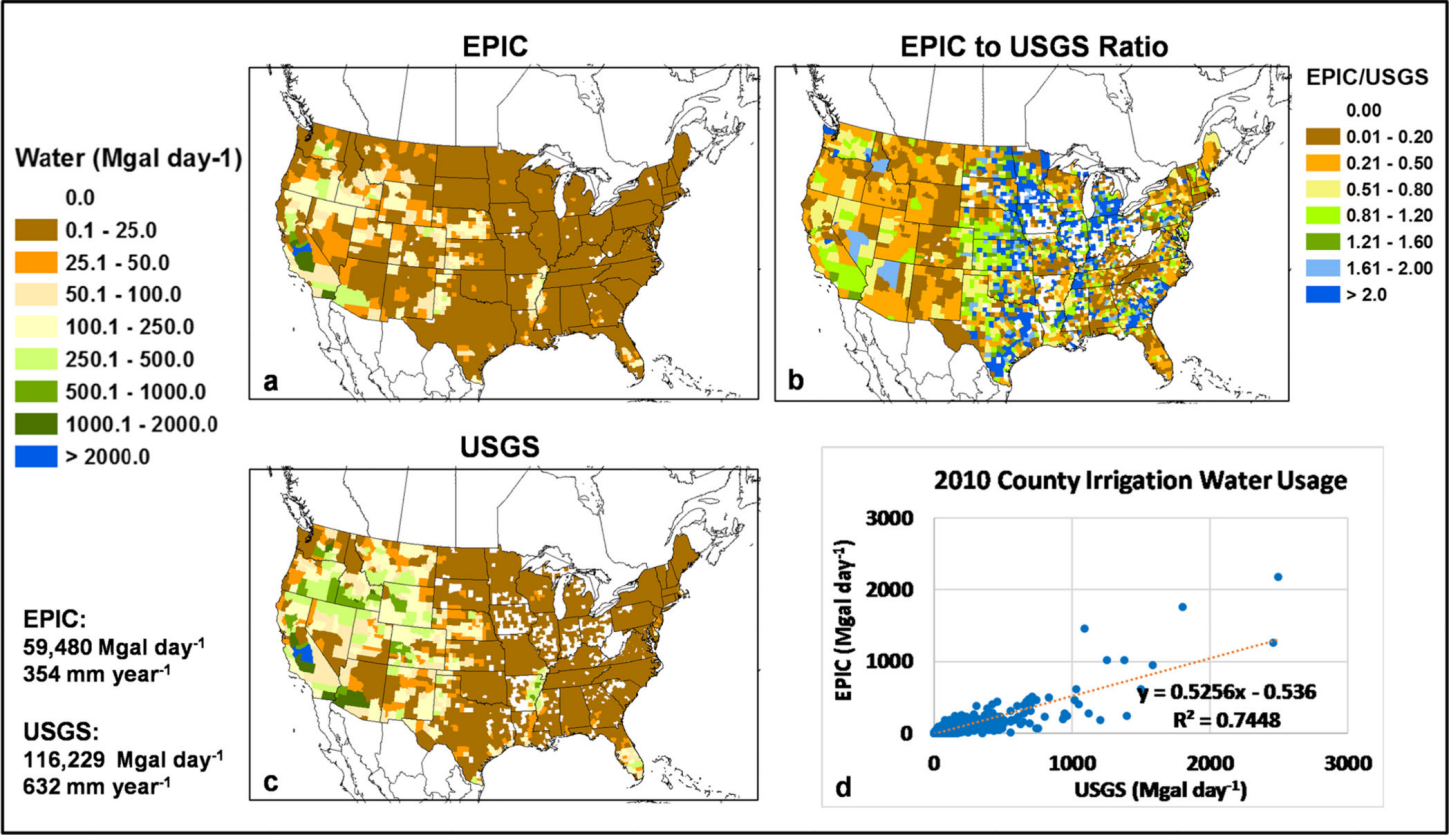
Comparisons of Environmental Policy Integrated Climate (EPIC)-simulated irrigation water withdrawals aggregated to the county with the U.S. Geological Survey (USGS) estimated use of irrigation water in 2010. The unit is at million gallons per day (Mgal/day).

Irrigation demand from agriculture production is the second largest water usage in United States despite the fact that only around 6% of all farmland (22.56 million hectares) is irrigated (Table S2). With intensive agricultural practices, the irrigated land is very productive with heavy fertilization. The simulated irrigation demand aggregated to the county is compared with the U.S. Geological Survey (USGS) water census data for 2010 (available every 5 years; Maupin et al., 2014) in Figure 7. Simulated irrigation demands (Figure 7a) show similar spatial patterns as the USGS census data (Figure 7c) with the highest demand in the San Joaquin Valley of California (CA). The east, except the lower Mississippi river valley and southern Florida, has relatively low water demands. Overall, simulated irrigation water domain wide from EPIC (59,480 million gallons per day [Mgal/day]) is about 51% of reported usage from USGS and the underestimation is further illustrated in the county irrigation water scatter plot (Figure 7d). It should be noted that the source of irrigation water varies between surface and ground water and the availability of surface water across the western United States varies with weather. The systems (e.g., gravity, sprinkler, and drip sprinkler) used to collect and distribute water were established historically, and the water laws vary from state to state (Benson et al., 1980). Thus, water application in the west is subject to rules (e.g., use it or lose it laws), while irrigation water in the Great Plains and eastward are likely to be from groundwater sources controlled by the farmer.

Difference is expected between EPIC-simulated demand and USGS water census given the complication and the assumed sprinkler system in EPIC along with WRF uncertainties, particularly in precipitation (Heath et al., 2016). Comparing with 2012 irrigated areas by methods at states reported in USDA NASS (2014), EPIC-simulated irrigation demands are close to USGS census in regions using sprinkler and drip methods such as California and from northern Texas to western Nebraska (Figure 7b). However, regions that predominantly use gravity irrigation such as the Mountain States from New Mexico northward to Montana tend to have much higher irrigation demand from USGS than EPIC. This is reasonable as gravity systems are much less efficient (use much more water) than the EPIC assumed sprinkler systems. Though many counties in the central States show EPIC water demands more than twice as high as USGS (deep blue in Figure 7b), the magnitude of the demand is relatively small. For improving water application and runoff on gravity regions, alternative irrigation parameters can be implemented in EPIC by methods of irrigation corresponding to regions.

#### 3.1.2. Yields

**Figure 8.**
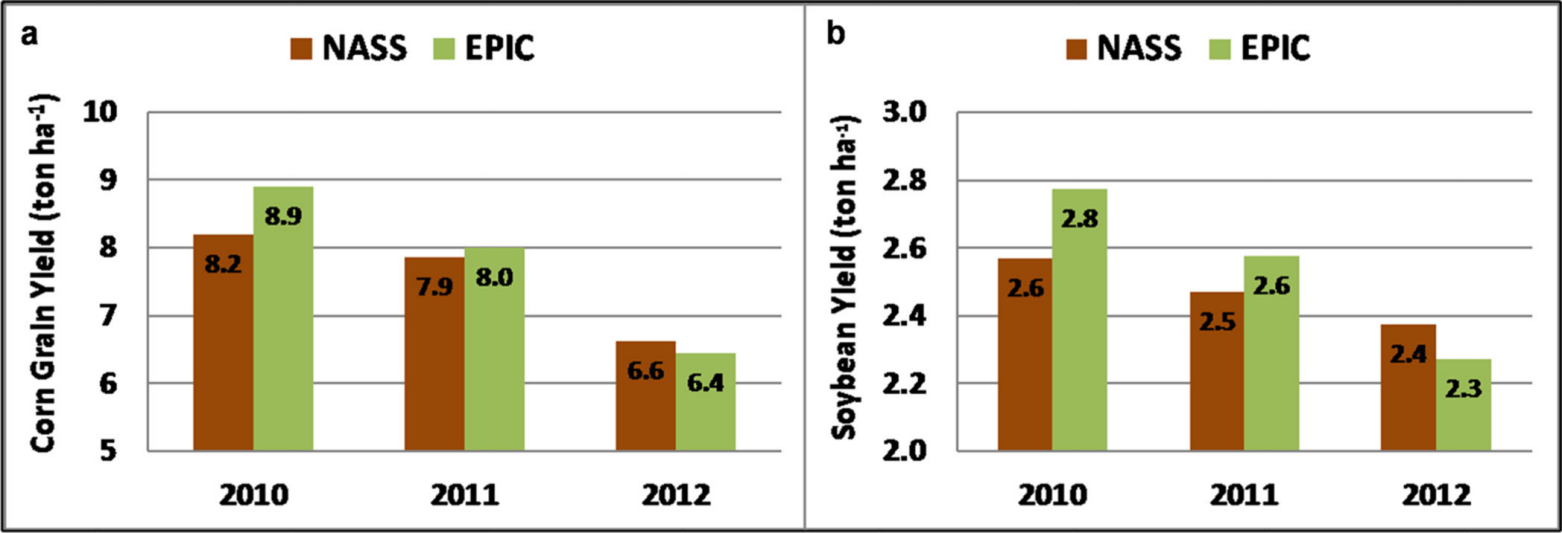
Simulated (a) corn grain and (b) soybean yields (ton/ha) domain wide in comparison with U.S. Department of Agriculture (USDA) National Agricultural Statistics Service (NASS) reports for 2010 to 2012.

**Figure 9.**
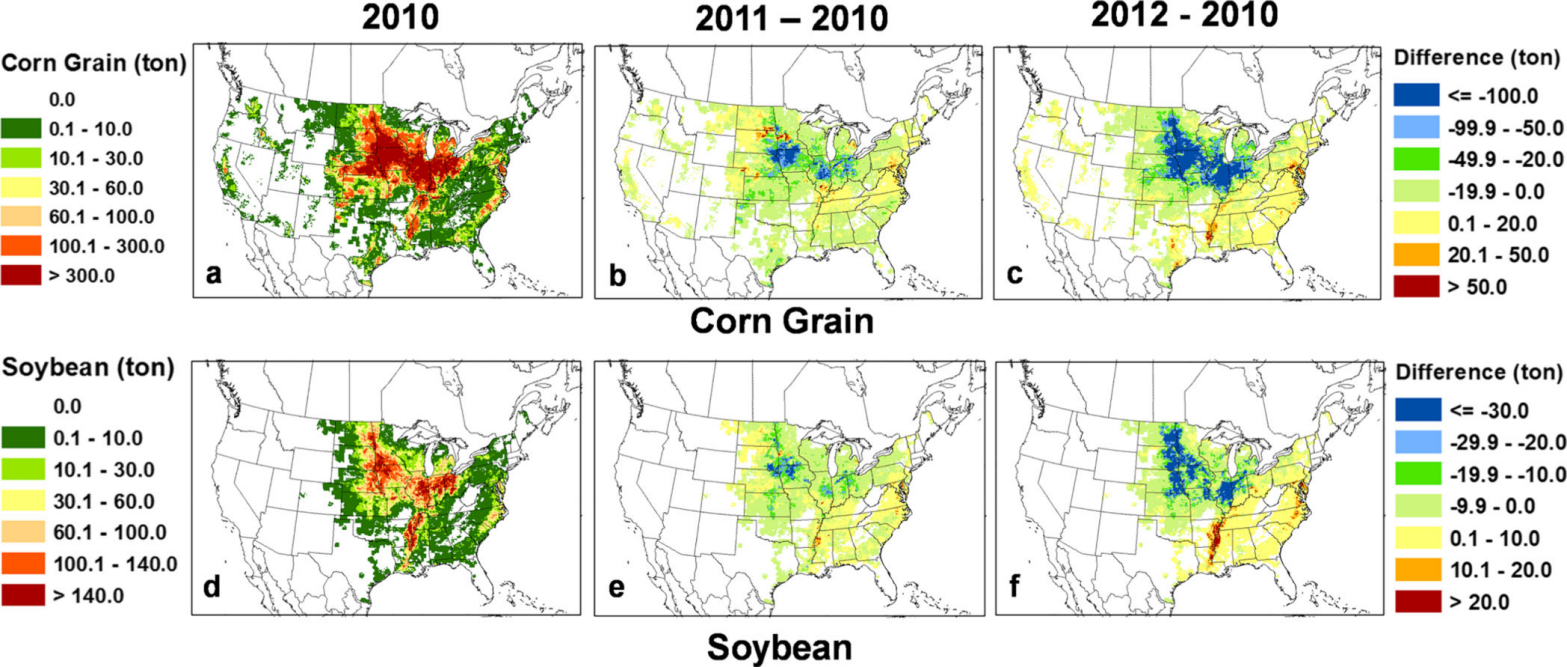
Simulated 2010 production (ton) for (a) corn grain and (d) soybean at the Community Multiscale Air Quality (CMAQ) 12-km grid cell and production difference for (b and e) 2011 and (c and f) 2012 from 2010.

Yields are most important output in cropping systems for performance evaluation. As corn grain and soybean are two dominant crops in United States (Figure 3), yield evaluation focuses on these two crops. Most of the two crops are planted on rainfed fields with 14.9% rainfed vs. 2.6% irrigated lands for corn grain and 13.6% rainfed vs. 1.6% irrigated fields for soybeans over the CONUS 2011. Domain-wide yield weighted by area is compared with USDA NASS report in Figure 8. Since plant transpiration is positively correlated with CO_2_ assimilations for plant growth, yield estimates show similar patterns to simulated ET (Figure 6), decreasing from 2010 to 2012 for both crops due to worsening dry conditions. EPIC yields, high in the first 2 years but low in the last year, are very similar to USDA NASS reports. Figure 9 shows spatial patterns of simulated production (ton) of corn grain (a) and soybean (d) for 2010 and production difference of 2011 (b and e) and 2012 (c and f) from 2010 at the domain grid cell. Major corn grain and soybean production are concentrated in the Midwestern Corn Belt region and along the lower Mississippi Valley. Year 2010 is the most productive year for both crops with many grid cells in the Corn Belt region having corn grain production more than 300 tons and soybean production more than 140 tons. The decreasing production for both crops in those high production grid cells is obvious from 2011 to 2012 (blue color in the difference plots). Corn grain production for 2011 is the lowest in Texas because of the worst 1-year period of drought in recent history. Both crops have the lowest production in the Corn Belt region for 2012 due to much reduced precipitation (Figure 5c) but higher production in the southeast, particularly the lower Mississippi Valley. Production change is relatively small outside the two concentrated regions due to low planting areas along with sufficient precipitation in the southeast and no water supply constraint with EPIC in the irrigated west. Overall, the system performs as expected over geographic regions given different meteorological conditions.

#### 3.1.3. N Budget With Fertilization

Budgeting N is an essential approach for evaluating the performance of the system and understanding the N cycle. Although the detailed partitions among different pathways simulated are beyond the scope of this paper, it is important to know N sources considered in this system and overall performance of N sources and output. N fertilization is the dominant external input to agriculture production. FEST-C EPIC simulates N fertilization in inorganic (synthetic such as NH_3_ and NO_3_ based) and organic (manure based) forms. Because FEST-C EPIC is simulated for CMAQ NH_3_ flux modeling on the agricultural field primarily with inorganic (or synthetic) N fertilization, the EPIC management is configured with a focus on inorganic fertilization. Only small amount of organic N fertilization is simulated in the current system for regions where inorganic fertilizer sales are less than the reported crop N demand. In CMAQ, NH_3_ emissions from concentrated animal feeding operations (CAFO) are estimated as point sources at the sites. Thus, the current system does not explicitly simulate the spread of manure on pastures around CAFO sites in order to avoid double counting of CAFO NH_3_ emissions in CMAQ. With this intentional fertilization management, the organic N fertilizer application on the average over the CONUS NLCD agricultural land for the 3 years is 3.6 N kg · ha^−1^ · year^−1^, which is lower than values reported in literature (Diebel & Zanden, 2009; Sobota et al., 2013), particularly for areas near CAFO sites with manure spreading.

**Figure 10.**
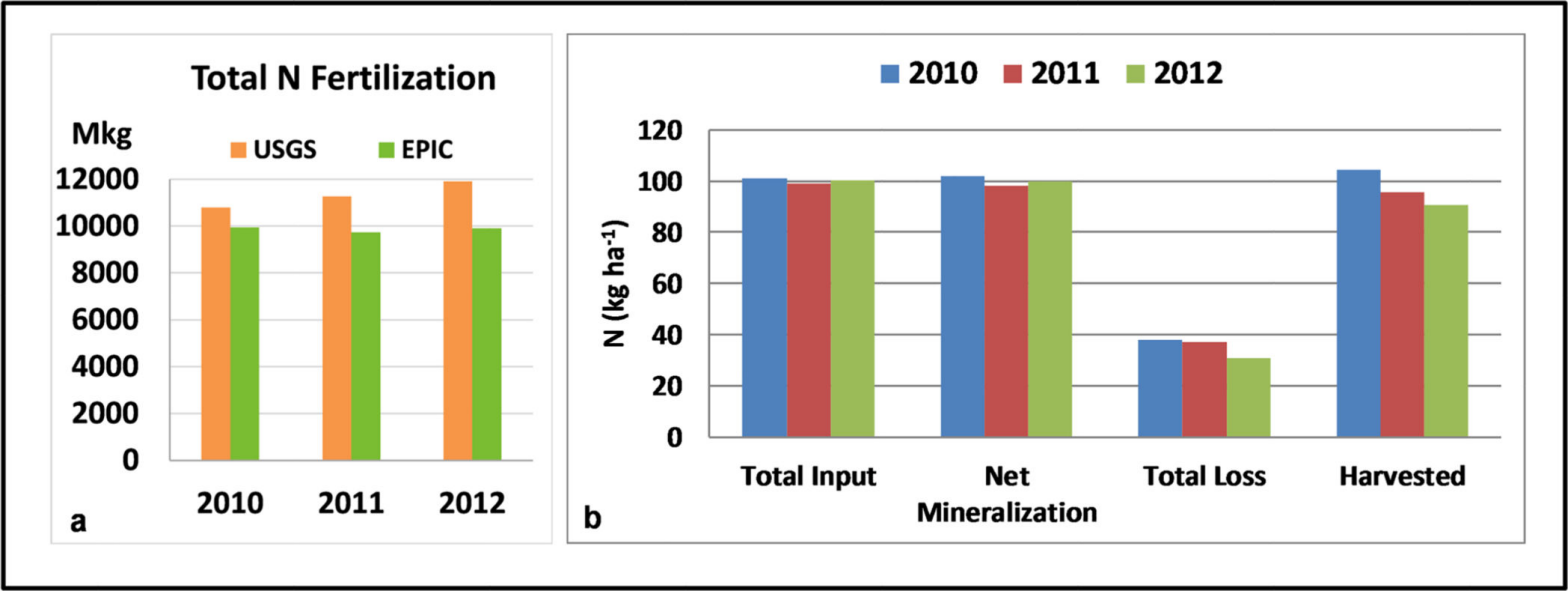
Domain-wide N input and output from the three different year simulations (2010 to 2012) with the comparison of domain-wide inorganic N fertilization between (a) EPIC-simulated and U.S. Geological Survey (USGS) reported and (b) the overall N budget with all N sources and pathways of N leaving the field. Total input includes the sources from fertilization, fixation, and deposition. Total loss includes the pathways from runoff (surface and subsurface with tile drainage), sediments, percolation, volatilization, and denitrification. Net mineralization is the internal N source from soil organic matter excluding immobilization and organic fertilization and Harvested is the N in harvested plants. The difference between total input + net mineralization and total loss + harvested represents the soil N pool at the yearend.

The overall N budget with fertilization in comparison with aggregated USGS county-level inorganic N reports (Brakebill & Gronberg, 2017) is displayed in Figure 10. Figure 10a shows the comparison of inorganic N fertilization between EPIC simulated and USGS reported (mainly from fertilizer sales). N fertilization reported by USGS for farm use shows a small increasing trend (around 5% each year); while EPIC-simulated N fertilization is quite similar for all three years, close to 10,000 million kg (Mkg) N/year (55.5 N kg · ha^−1^ · year^−1^ on average) and around 13% on average below the USGS average (63.8 N kg · ha^−1^ · year^−1^). Traditionally, fertilizer is applied to the field before or during planting and in the early plant growing season (Cassman et al., 2002). However, farmers never know for sure how much N fertilizer is required given uncertainty in precipitation and in SOM (Stewart & Lal, 2017). For ensuring high yield, farmers often apply enough fertilizer with the expectation that the growing season weather, particularly precipitation, will be normal in order to avoid N limitation which is more controllable than precipitation. Thus, most fertilization is completed before the growing season (to avoid plant damage by machines) when the influence of weather is crucial. Given the crop N demand-based fertilization scheme, the simulated inorganic N fertilization is expected to be lower (or more efficient) than USGS reported farm use. With the same spinup and the realism of the management, the simulated fertilization is similar among the 3 years and is comparable to the USGS value as demonstrated despites different weather conditions. On average, the total external input, including fertilization (59.1 N kg/ha), fixation (31.8 N kg/ha), and deposition (9.3 N kg/ha), is 100 N kg · ha^−1^ · year^−1^. It is about 24% lower than the estimate (131.1 N kg · ha^−1^ · year^−1^, USDA national value divided by FEST-C agricultural land) in the USDA Conservation Effects Assessment Project (CEAP) report (USDA NRCS, 2017), which has fertilization rates based on survey information from farmers. The underestimation is mostly from low inorganic and organic fertilization simulated in this system due to the intended model configuration. Despite the underestimation, the magnitudes of total simulated inorganic fertilization, fixation, and deposition domain wide seem to be within the range reported in literature as summarized by Sobota et al. (2013).

The net mineralization (excluding N immobilization and organic fertilization) is the internal source of N from SOM and is often overlooked because of the complexity and many unknowns. In top-down approach N budget studies (e.g., inventory-based approaches using yield, fertilizer sales, and other information), it is often assumed that SOM along with mineralized N is in a stable state (e.g., Zhang et al., 2015). Many biogeochemical processes in SOM buildup and N mineralization are influenced by weather/climate and soil properties (Stockmann et al., 2013). Meanwhile, the model configuration also influences the dynamics of N cycling. Thus, it is important to understand the contribution of the internal N from SOM in this system because both external and internal N contributes to the pools of plant uptake and loss pathways. Agricultural production over the long term does degrade soil nutrient contents at a level depending on management practices. The spinup of the system is configured with conservation tillage and minimal erosion resulting in high SOM and N mineralization contributing to the source pool almost as much as the total input domain wide. The net mineralization (99.9 N kg · ha^−1^ · year^−1^ on average) seems to be in the range of values estimated in field experiments (Campbell et al., 2008; Carpenter-Boggs et al., 2000) and in processed-based models (e.g., Smith et al., 2008). Assuming all external N goes to the harvest and loss pools, the contribution from net mineralization to the pools is 32.4 N kg · ha^−1^ · year^−1^ on average, which is likely to be on the high side. Because the system is designed to simulate production for a specific weather year, this high N contribution from SOM does not realistically represent severe soil degradation on the agricultural land. Instead, it shows the dynamical response of the system to the relatively low fertilization, which results in high contribution from net mineralization to meet crop N demand.

In contrast to relatively stable N external and internal pools, N total loss and N in harvested plants are much more sensitive to year-specific weather conditions and they demonstrate a decreasing trend from 2010 to 2012 (Figure 10b) following the worsening drought in the west. The total loss includes all N losses through the pathways of surface and subsurface (with tile drainage) runoff, sediments, percolation, volatilization, and denitrification. The total loss simulated by EPIC is 35.4 N kg · ha^−1^ · year^−1^ on average, which is similar to 38.6 N kg · ha^−1^ · year^−1^ in the CEAP report (USDA NRCS, 2017). The loss is 19%, 19%, and 16% of the total external and internal N input for years of 2010, 2011, and 2012. The dominant N output from the field is from N removal in crop yield at harvest. On average, the system estimates 97.0 N kg · ha^−1^ · year^−1^ removal rate in harvest, which is also very close to 95.0 N kg · ha^−1^ · year^−1^ estimated in the CEAP report. The N in harvested plants accounts for 51%, 49%, and 45% of the total external and internal input. With much reduced yield as demonstrated in Figure 8, the year of 2012 has the lowest N harvested as well as the lowest N lost to the environment due to the extremely dry conditions. Thus, more N is left in the field in the least productive year, while N leaving the field is the highest for the most productive year of 2010. The N remaining in the agricultural soil at the yearend is 60.3, 64.0, and 78.3 N kg/ha for the years of 2010, 2011, and 2012 and on average is 67.6 N kg · ha^−1^ · year^−1^.

**Figure 11.**
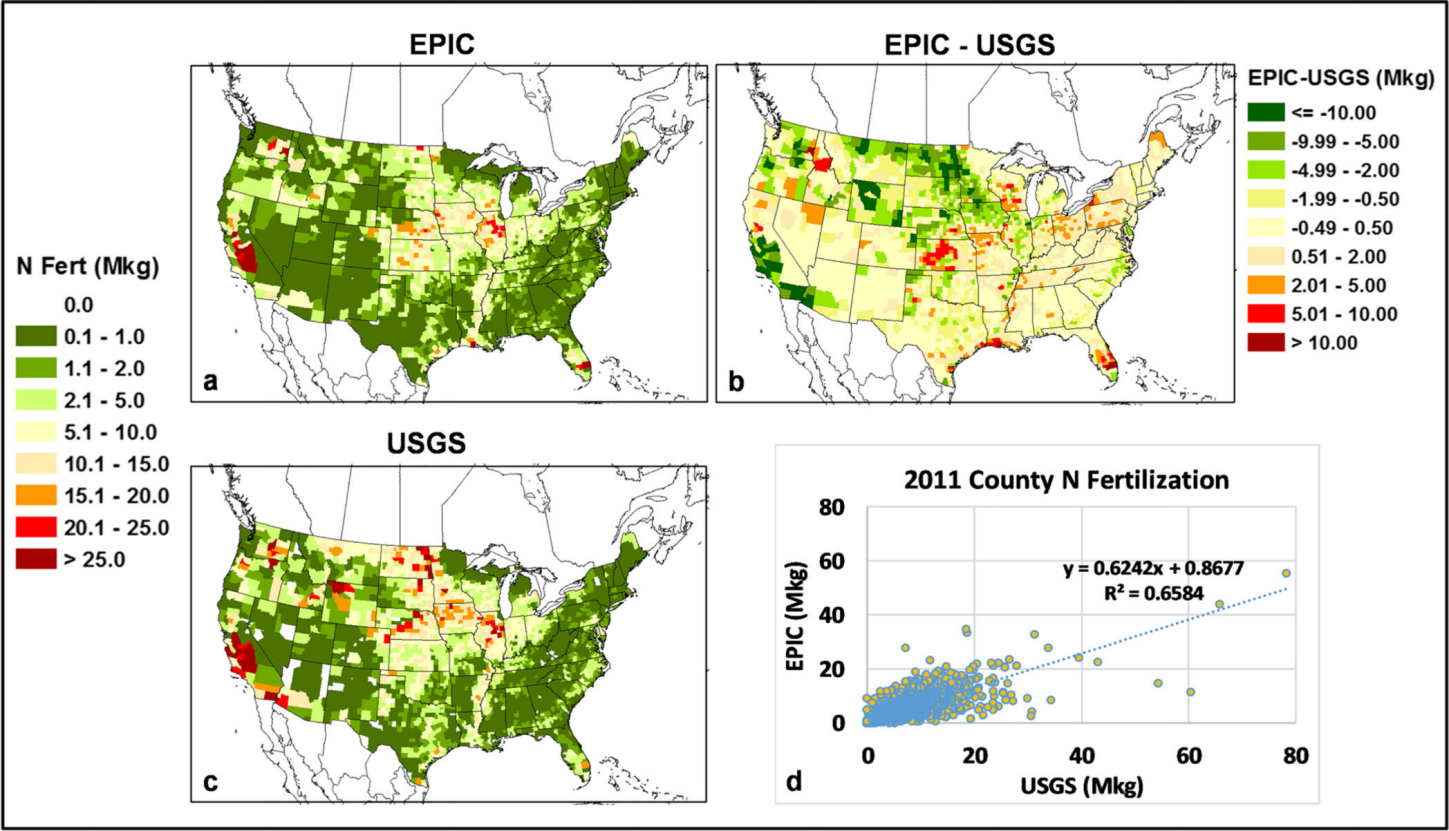
Comparisons of EPIC-simulated N fertilization aggregated to the county with the U.S. Geological Survey (USGS) estimated use of farm N fertilization for the year of 2011. The fertilization amount is displayed in million kg (Mkg) N/year.

The simulated inorganic N fertilization aggregated by county is compared with the USGS data spatially for 2011 in Figure 11. The overall underestimation of EPIC-simulated N fertilization is also demonstrated by the county fertilization scatter plot (Figure 11d). Following the agricultural land distribution in Figure 2, simulated N fertilization (Figure 11a) shows similar spatial patterns as the USGS data (Figure 11c) with the highest demand from the Central Valley in CA. The USGS data show much higher fertilizer use in some counties, particularly from the northern Plains states (North and South Dakota, Nebraska), northwestern states (Wyoming, Oregon), and southwestern states (CA and Arizona). For instance, Park County in the northwestern corner of Wyoming has USGS 30.66 Mkg but EPIC 2.49 Mkg and Imperial County in the southeastern corner of CA boarding Mexico has USGS 54.34 Mkg and EPIC 14.79 Mkg. Monterey County on the Pacific coast of CA has the biggest discrepancy with USGS 60.47 Mkg and EPIC 11.40 Mkg. The USGS fertilizer use in those counties is very disproportionate to their relatively small agricultural land reported in the USDA NASS 2011 Crop Data Layer (CDL; Boryan et al., 2011). The big discrepancy is likely caused by fertilizer sales to farmers from surrounding counties as fertilizer sales are related to not only fertilization demand but also store locations and prices. For some counties in Kansas, Wisconsin, southern Louisiana (LA) and Florida (FL), and southeastern Washington into Idaho, simulated fertilization demands are particularly high. The EPIC as configured here tends to simulate high yield along with high fertilization for small grains such as barley, oats, and wheat. Thus, counties in Kansas and the areas near the Washington and Idaho border, which are predominantly wheat, tend to have high simulated fertilization. All crops not explicitly considered in FEST-C (such as sugarcane, idle land, vegetables, and orchards) are categorized into rainfed And irrigated Other Crop types (37, 38 in Table S1). Considering plants with varying fertilization demands in the two Other Crop types, FEST-C EPIC is configured to treat Other Crop like corn with relative high fertilization demand. Some of these Other Crop plants such as fruits and vegetables in CA likely have higher fertilizer needs contributing to the underestimation of EPIC as displayed while other plants in Other Crop types may have lower fertilizer needs. For instance, the three counties in southern LA (Vermilion Parish) and FL (Palm Beach and Hendry counties) with high EPIC fertilization (Figure 11b) also have very high Other Crop type areas including sugarcane, idle land, and oranges based on the 2011 CDL. This indicates limitations in simulating agriculture production using the Other Crop categories lumped with different production types and idle land.

In summary, the overall N budget with simulated fertilization performs as expected given the different weather conditions along with the typical regional management practices prescribed. Even though limitations and issues (e.g., wheat and Other Crop issues) exist in different regions, simulated fertilizer demands do follow agricultural land distribution well at the county level in comparison with the USGS sales-based fertilization. The spatial and temporal information in the N budget including all sources and loss pathways, which is often missing in inventory-based N budget studies, is particularly valuable. In addition, it is clear that specific-year weather plays a dominant role in dictating how much N leaves the field through either the loss pathways or harvesting, while the weather has much less impact on overall N input. Given the unpredictable nature of growing season weather and changing climate, it is truly a challenge to both agricultural and environmental communities to ensure food production while reducing N loss to the environment. With this integrated system, consequences of different management practices for maintaining and enhancing SOM and reducing runoff and soil erosion along with different fertilization strategies can be explored and evaluated under different weather/climate and N deposition scenarios. The allocation of fertilizer by type, timing, and application method in the EPIC management files offers an opportunity to estimate the physiological processing of different forms of fertilizer. Ammonia, nitrate, and organic N are applied and processed differently and at different times allowing users to estimate the impact of form, timing, and method of application on crop production as well as air and water quality.

### 3.2. Integrated Atmosphere

CMAQ (version 5.0) has incorporated N fertilizer application information and soil conditions from EPIC output for bidirectional NH_3_ modeling as demonstrated by Cooter et al. (2012). Since then, the system has gone through many updates and advances to the current release of CMAQv5.3 (https://github.com/USEPA/CMAQ). Different from the previous bidirectional NH_3_ approach using the fertilization information to simulate soil NH_3_ in CMAQ, a new approach of bidirectional flux modeling, which directly uses EPIC-simulated soil properties including soil NH_3_ concentration (Pleim et al., 2019), is implemented in the current release of CMAQ. The new approach directly follows the bidirectional flux box model based on field studies demonstrated by Pleim et al. (2013) and has additional updates to some key parameterizations. Air quality simulations are conducted using CMAQ v5.2 updated with the new bidirectional approach (Bidi) for two evaluation scenarios with/without bidirectional NH_3_ flux modeling (Bidi vs. Base) for the same CMAQ CONUS domain. CMAQ along with needed 2011 WRF meteorology is configured with the physics options typically used for EPA air quality studies (Appel et al., 2013; Gilliam & Pleim, 2010; Hogrefe et al., 2015). Detailed information on the CMAQ configuration, processed NEI 2011 emissions, and other needed input including boundary conditions is presented in the study by Appel et al. (2017). The extracted CMAQ-ready daily NetCDF capacity, depth, moisture, and soil NH_3_ content.

**Figure 12.**
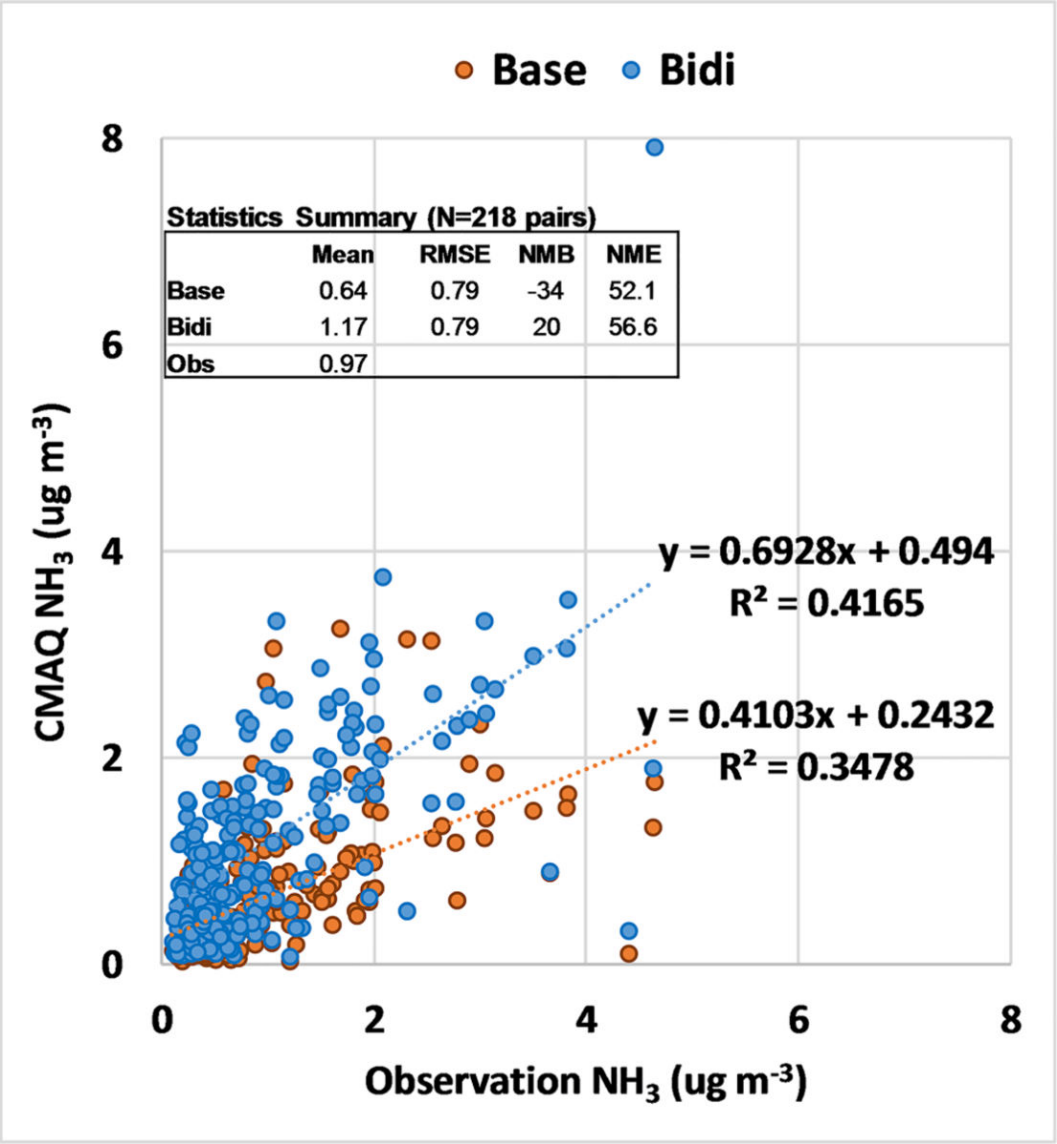
Scatter plot with overall statistical metrics for the simulated surface-layer gas-phase NH3 concentrations from the Base and Bidi scenarios against Ammonia Monitoring Network (AMoN) observations over the growing season from 1 April to 30 September 2011.

The simulated gas-phase NH_3_ concentration is compared with the ambient gas-phase NH_3_ measurements from the Ammonia Monitoring Network (AMoN) under the National Atmospheric Deposition Program (NADP). AMoN NH_3_ observations are made on a 2-week accumulated average basis at 54 sites over CONUS for 2011 (NADP, 2012), and the analysis only includes the data flagged as valid. Figure 12 shows the scatter plot of valid measurements with simulated NH_3_ concentrations from the Base (brown) and Bidi (blue) scenarios over the growing season from 1 April to 30 September 2011. Using 2011 NEI NH_3_ emission estimates from the agricultural land fertilization (U.S. EPA, 2015), the Base scenario shows overall underestimation of surface-layer NH_3_ concentrations with mean 0.64 (μg/m^3^) and normalized mean bias −34% in comparison with the observed mean 0.97 (μg/m^−3^). Driven by soil physical and chemical properties from EPIC on the agricultural land, the Bidi scenario exhibits a tendency of overestimation with a mean of 1.17 (μg/m^−3^) and normalized mean bias of 20%. Despite its slightly higher normalized mean error, the simulated NH_3_ concentration from the Bidi scenario performs better overall including a higher correlation (higher R-square) with the observations.

**Figure 13.**
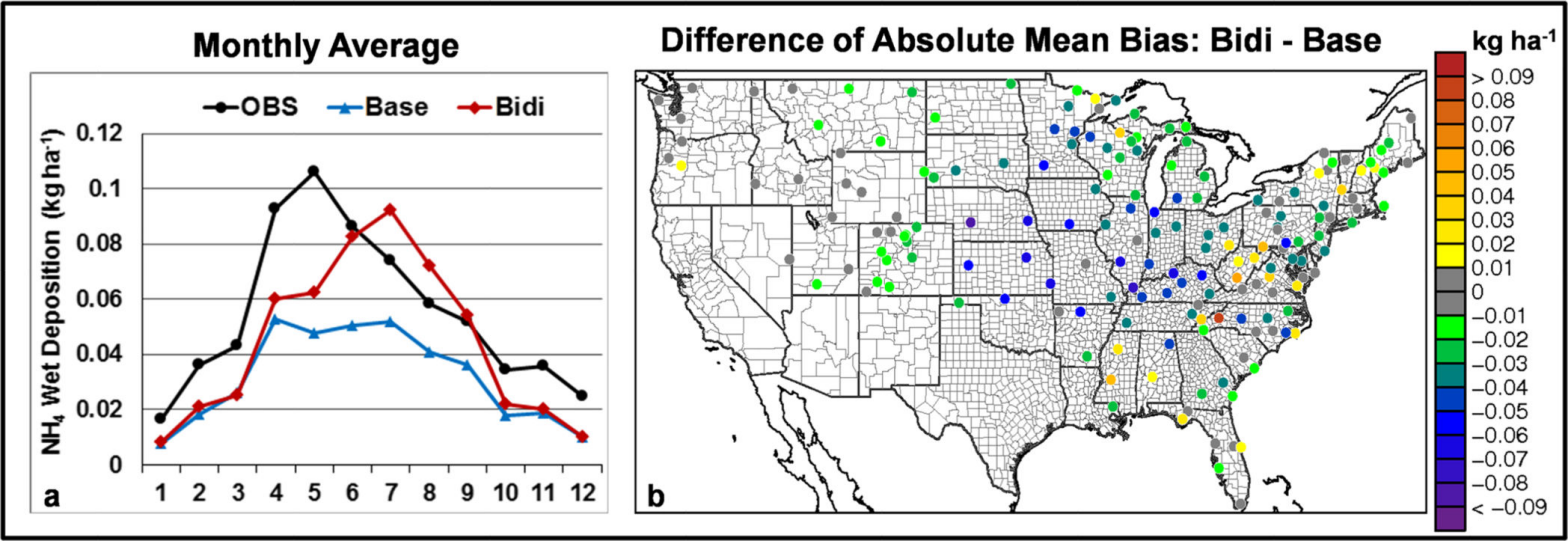
(a) Comparison of the monthly mean NH_4_^+^ wet deposition between the Base and Bidi scenarios against valid measurements from 231 NADP NTN sites and (b) difference of absolute mean bias plot (Bidi – Base) of the wet deposition at 181 NTN sites for the period from 1 April to 30 September 2011. Note that only sites with more than 50% of data coverage are included in the spatial plot.

Simulated NH_4_^+^ wet deposition is evaluated against the NADP National Trends Network (NTN) measurements that are made on a weekly accumulated basis for 2011 (NADP, 2012). Figure 13 shows the monthly average comparison between the simulated and the observed deposition (a) and the spatial plot for difference of absolute mean bias (DAMB) between the Bidi and Base deposition over the growing season from 1 April to 30 September in 2011 (b). The underestimation is apparent, particularly for the Base scenario. The two scenarios have very similar performance outside of the growing season from November to March but quite different performance during the growing season with much reduced low bias in the Bidi scenario (Figure 13a). The observations show the peak deposition in May, while the Bidi shows a peak later in July. The Base, without an obvious peak month, underestimates the deposition every month, while the Bidi scenario seems to be too high in July and August. The much-improved performance during the growing season with the Bidi configuration is also demonstrated by reduced biases at many NTN sites (negative values in green and blue colors) in the spatial plot of DAMB (Figure 13b). Although a few sites in limited areas (e.g., West Virginia, western North Carolina, and Mississippi) exhibit higher bias in the Bidi scenario, the average DAMB of all sites is −0.017 kg/ha with obvious bias reduction from the integrated CMAQ with EPIC soil information in the Central and northern Plains States where the agricultural land production is intensive.

NH_3_ and NO_x_ emitted from fertilized agricultural soil influences atmospheric compositions through ammonium aerosol (ammonium nitrate, ammonium sulfate, and ammonium bisulfate) formation and ozone production in CMAQ. The evaluation of the CMAQ gas-phase NH_3_ and NH_4_^+^ wet deposition above demonstrates that EPIC-derived spatial and temporal information on agricultural land helps improve estimation of land-atmosphere NH_3_ fluxes and consequently the simulated air quality. The performance of EPIC simulations directly influences NH_3_ flux modeling in CMAQ through the amount of NH_3_ in soil available for volatilization. The NH_3_ content along with other soil properties in agricultural fields is a direct result of many complicated soil processes interrelated to biogeochemistry, hydrology, weather/climate, N deposition, and management practices including fertilization. Those interdisciplinary processes involve many parameterizations, which have uncertainties requiring field experiments, research, and improvements continuously (Izaurralde et al., 2017; Brilli et al., 2017). In this integrated system, improved atmospheric WRF/CMAQ benefits EPIC agricultural production simulations with more accurate weather and N deposition. EPIC with better atmospheric input likely results in better agricultural soil representation, which in turn helps reduce the uncertainty in CMAQ NH_3_ flux modeling.

### 3.3. Integrated Hydrology and Water Quality

**Figure 14.**
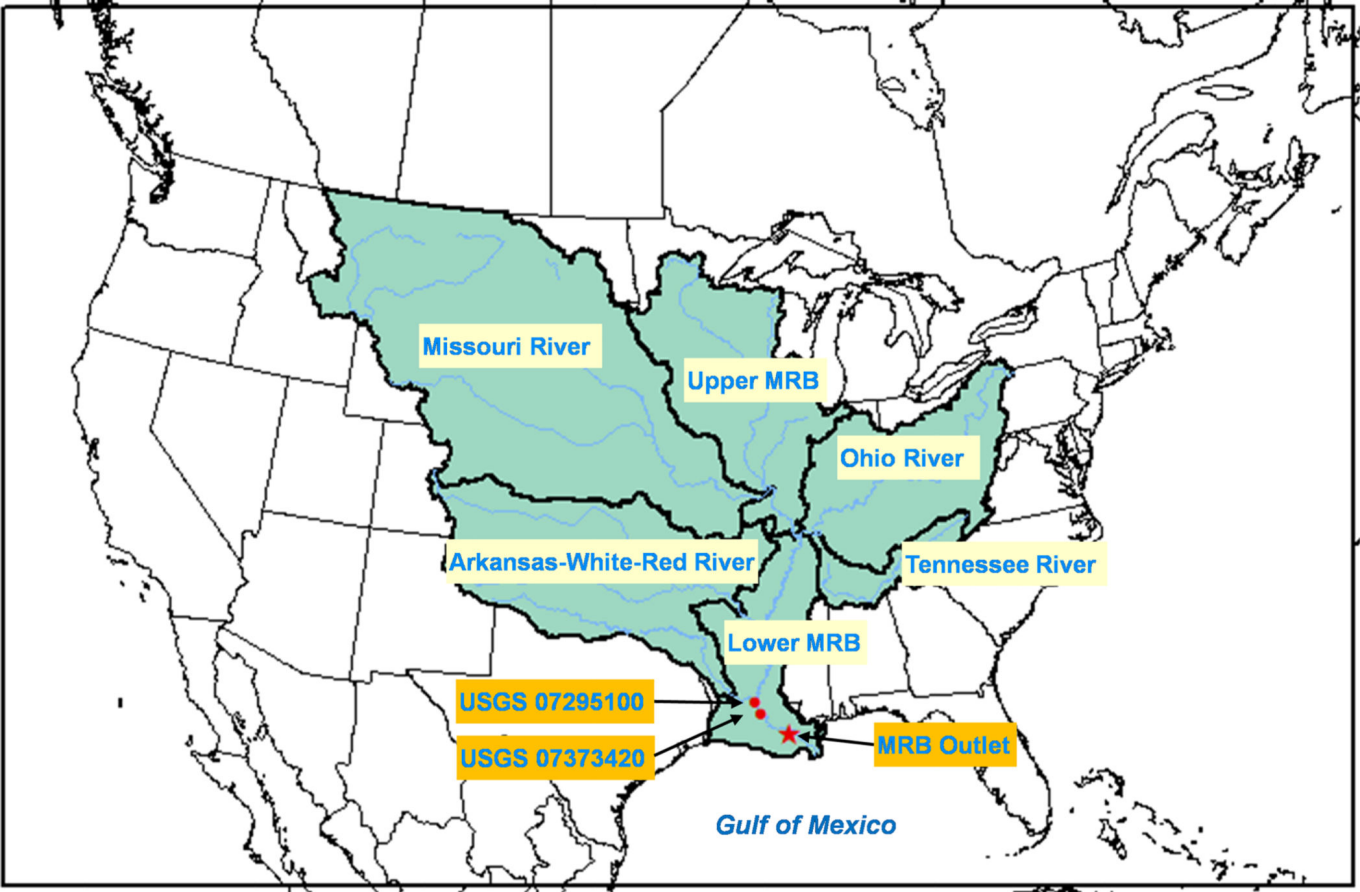
The Mississippi River Basin (MRB) in the Community Multiscale Air Quality (CMAQ) conterminous United States (CONUS) 12-km domain. MRB comprises six two-digit hydrologic unit code subbasins. Two U.S. Geological Survey (USGS) monitoring stations used in evaluation from the Lower MRB near the outlet (red star) to the Gulf of Mexico are displayed as red dots.

Integrated SWAT with EPIC-WRF-CMAQ using FEST-C is described and demonstrated by Yuan et al. (2018) with an application to the MRB. Using the EPIC simulations conducted in FEST-C v1.3 and WRF/CMAQ-simulated weather and N deposition for each year from 2002 to 2010, they show that the integrated SWAT (IMS-SWAT) improves estimations of stream flow and dissolved N loadings to the GOM over the period. As EPIC in the current release has many changes, particularly in C-N cycles and hydrology processes, the performance of IMS-SWAT is further demonstrated using the evaluated three-year EPIC results from the current release. The Mississippi River is the largest river in North America draining 41% of the CONUS and flowing over 2,300 miles through the U.S. heartland to the GOM (Figure 14). While leading the world in the agricultural production and providing water to industry and millions of people, the basin has been facing a critical challenge with nutrient pollution in recent decades. Massive amounts of nutrients escape from agricultural lands, sewage treatment plants, and other sources into rivers and groundwater (Alexander et al., 2007; Burow et al., 2010) contributing to the seasonal hypoxia in the northern GOM and posing threats to human health and ecosystem services. Large-scale integrated assessment tools are clearly needed to evaluate nutrient sources, fate, and transport in a more holistic manner. The ability of the FEST-C system to facilitate multimedia connections is demonstrated through application to the MRB.

SWAT with land and stream processes simulates water runoff, loadings of sediments, and other constituents such as dissolved N (e.g., NO_3_^−^ and NO_2_^−^) with the consideration of point sources (e.g., sewage treatment plants) and agricultural production processes (Arnold et al., 2012; Gassman et al., 2007). The agricultural production processes are switched off in the IMS-SWAT configuration; instead, surface and lateral runoff of N-P and water and sediment loads from EPIC are directly delivered to each eight-digit HUC watershed outlet based on the USDA Conservation Effects Assessment Project study approach (Wang et al., 2011; White et al., 2014). The web-based Hydrologic and Water Quality System (HAWQS v1.0, https://epahawqs.tamu.edu/; Yen et al., 2016), which is developed at TAMU with the support from the U.S. EPA, is used to prepare SWAT input files for 821 eight-digit HUC watersheds in MRB. Because HAWQS is limited to CONUS, the very small portion of MRB in Canada is not included in the simulation. While SWAT default parameters in HAWQS have some preliminary calibration, no additional calibration is conducted for the integrated simulation. SWAT is configured with physical options and input parameter files the same as applied in the study by Yuan et al. (2018). The simulated results are compared with measurements from two USGS stations near the MRB outlet (red dots in Figure 14), which are selected for evaluation in their study.

**Figure 15.**
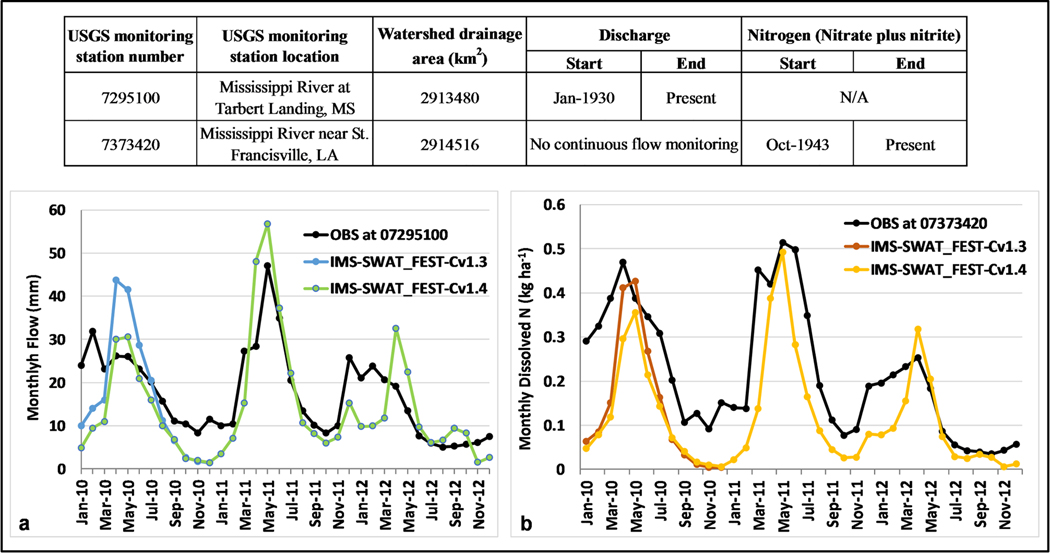
Comparisons of (a) simulated monthly stream flow (mm) from the integrated Soil and Water Assessment Tool (SWAT) by Fertilizer Emission Scenario Tool for CMAQ (FEST-C) V1.4 (IMS-SWAT_FEST-Cv1.4) and FEST-C V1.3 (IMS-SWAT_FEST-Cv1.3) with the observation at USGS monitoring station 07295100 and (b) dissolved N (kg ha^−1^) with the measurement at U.S. Geological Survey (USGS) monitoring station 07373420 over the drainage area from 2010 to 2012. USGS monitoring station information is displayed in the top of the figure. Integrated SWAT with FEST-C V1.3 from Yuan et al. (2018) has only 1 year (2010) overlapping with the integrated SWAT simulation with FEST-C V1.4.

Figure 15 shows the simulated monthly streamflow (a) and dissolved N (b) in comparison with measurements at the USGS stations with the station information displayed in the top of the figure. The integrated SWAT using FEST-C V1.3 EPIC in Yuan et al. (2018) is also displayed for overlapped simulation year 2010 to show the influence of updated EPIC on SWAT. The integrated SWAT from both versions shows similar performance for 2010, with updated EPIC resulting in reduced high bias of simulated peak monthly stream flow and increased low bias for dissolved N. The improved hydrology in the updated EPIC helps SWAT peak flow estimation, and the lower peak N loading from the new version is consistent with high bias reduction of N fertilization from much improved N cycling in the current release (Ran et al., 2018). The USGS observed monthly streamflow shows peak flows in winter and spring seasons and low flows during the fall. The simulated flow at the MRB outlet shows distinct peaks in April and May with overestimation for all three years, while the low flow in the fall shows some underestimation. The IMS-SWAT peaks compare well with the observation in May for 2011, which is the wettest year (Figures 5 and 6) with the highest peak in both the model and observation. The 3-year average monthly flow observed at the USGS station 07295100 is 17.2 mm, and simulated from IMS-SWAT is 14.3 mm. The simulated monthly dissolved N peaks compare well with the observed values at the USGS station 07373420. Following the simulated streamflow, the dissolved N peaks in May and drops to the lowest values in the fall. For most months, IMS-SWAT underestimates the monthly dissolved N with the average monthly dissolved N 0.12 kg/ha compared with the observed value 0.22 kg/ha. Despite differences, the simulated monthly streamflow and dissolved N loadings at the MRB outlet show similar seasonal trends as observed.

Differences between simulated results and observed values in this integrated hydrology and water quality modeling could originate from many sources. The uncertainty in daily weather data from WRF, particularly precipitation amount and location, influences streamflow directly. The retrospective WRF-simulated weather often performs well for near-surface temperature, moisture, and windspeed with surface observation assimilation (Gilliam & Pleim, 2010). However, modeling precipitation at right time and location with correct magnitude is always a challenge due to complicated cloud processes. Since simulated precipitation in WRF configured with typical physics options used for EPA air quality studies tends to have high bias (Ran et al., 2015), improving precipitation simulation such as using lightning data assimilation (Heath et al., 2016) will help reduce some overestimation of peak streamflow. Meanwhile, uncertainties associated with SWAT groundwater recharge and snowmelt components along with omitted irrigation demand and other uses could all contribute to overestimation in the peak season and underestimation in low flow seasons (Yuan et al., 2018). For dissolved N loadings, uncertainties associated with N sources such as N fertilization, N deposition, point sources from sewage treatment and animal husbandry, and other sources such as urban runoff and legacy nutrient accumulation all influence the performance of the nutrient simulation. Some underestimation of the N loadings likely comes from the configured EPIC fertilization scheme, which results in low organic and inorganic fertilization. In addition, since FEST-C EPIC simulates limited production types (42 types) with regional management representation, discrepancies are expected at finer scales such as at watersheds and farms. However, there is not currently a database of management, tillage, and fertilization practices by fertilizer type, timing, and application methodology for all crops produced in the basin. Improving FEST-C EPIC will clearly influence the integrated SWAT performance as demonstrated in the limited 1 year overlapping comparison. Besides the influence of EPIC and WRF/CMAQ, the configured IMS-SWAT also has limitations due to no calibration and relatively short-period modeling for the demonstration purpose. As EPIC, WRF/CMAQ, and SWAT along with FEST-C are continuously being updated and advanced by the modeling communities, the integrated system will improve.

## 4. Conclusions and Future Work

This paper describes a regional-scale integrated modeling system that includes agriculture EPIC, atmosphere WRF/CMAQ, and hydrology and water quality SWAT models. The Java-based interface FEST-C system, which includes EPIC adapted to regional applications, is the central piece of this integrated system. The system was initially developed for integration with WRF/CMAQ, which provides weather and N deposition to agricultural simulations and EPIC-simulated soil properties with N fertilization information for CMAQ bidirectional NH_3_ flux modeling on agricultural land (Cooter et al., 2012). Over the years, the system has gone through many enhancements and changes up to the current release of FEST-C V1.4 (Ran, Cooter, Yang, Benson, et al., 2018). The enhancement of the system with the SWAT integration capability is a key feature of the current release. With FEST-C V1.4 tools, large-region watershed hydrology and water quality simulations can be conducted using SWAT integrated with EPIC and WRF/CMAQ results (Yuan et al., 2018). In addition, the system enables users to generate land use data with 42 production type fractions needed for EPIC and WRF/CMAQ bidirectional NH_3_ modeling from any one of the three NLCD data sets available (2001, 2006, and 2011) for any WRF/CMAQ domain over CONUS. The system is released with two 5-year average daily N deposition data sets from 2002 to 2006 and 2006 to 2010, processed from CONUS CMAQ simulations (Appel et al., 2013; Zhang et al., 2019), for reflecting N deposition changes due to tightened standards by U.S. EPA under the CAA on NO_X_ emissions before 2007 (Simon et al., 2014). Thus, the influence of N deposition on agricultural production and water quality can be explored by selecting either of the processed 5-year average N deposition data or year-specific N deposition input from WRF/CMAQ under consistent atmospheric conditions.

The modeling system presented in this paper enables integrated examinations of multimedia connections of N sources, fate, and transport over a large region for ensuring food security while sustaining the environment. To demonstrate the system capabilities, agricultural production simulations are conducted for 3 years (2010 to 2012) individually over a CMAQ CONUS domain using FEST-C EPIC integrated with WRF/CMAQ. Domain-wide simulated water and N budgets and yields respond to different-year weather conditions as expected. Though simulated irrigation demand for 2010 is about half of reported usage from USGS water census, the simulated spatial variability in irrigation demand is expected given the assumed use of only sprinkler system across the CONUS. Despite the tendency to be lower than the reported usage, simulated demand-based inorganic fertilization for 2011 at the county level follows the agricultural land distribution better than USGS sales-based usage. Integrated air quality modeling is demonstrated for 2011 over the same domain using CMAQ configured with a bidirectional NH_3_ modeling approach that directly uses EPIC-simulated soil properties including soil NH_3_ content (Pleim et al., 2019). With spatially and temporally better represented soil NH_3_ information from EPIC and consequently improved representation of land-atmosphere NH_3_flux, CMAQ shows better performance in simulating gas-phase NH_3_ and NH_4_^+^ wet deposition in comparison to the traditional approach based on NH_3_ emission estimates solely from fertilizer sales data. Integrated SWAT with EPIC and WRF/CMAQ results through FEST-C are applied to the MRB eight-digit HUC watersheds for demonstrating watershed hydrology and water quality simulations. While improved FEST-C EPIC influences the SWAT performance, simulated monthly streamflow and dissolved N loadings near the outlet to the GOM demonstrate similar seasonal patterns as observed given the modeling constraints and the complexity of the large basin.

The agriculture, atmosphere, and hydrology models integrated in FEST-C are discipline-specific comprehensive computer tools, which have long been used by researchers and policymakers around the world. The modeling tools have uncertainties as demonstrated because of many unknowns in complex and interrelated processes, particularly in soils (Brilli et al., 2017; Stockmann et al., 2013), influenced by natural and anthropogenic sources. In addition, there are limitations in FEST-C EPIC due to its configured 42 agricultural production types and associated regional management representation. Some of the uncertainties with EPIC are due to simulation of “Other Crops” as corn given high variations of fertilizer needs by different fruits, vegetables, and orchards. Thus, improvements are needed for areas dominated with Other Crops types, which show much too low or high fertilization demand. For improving simulated irrigation demand, different irrigation methods can be implemented regionally according to USDA NASS reports. As accurately representing land use, soil, and management practices is crucial, updating those components in FEST-C with improved information such as CDL, better soil data, and recent census information will all benefit the system. Since phosphorus is also an important nutrient influencing plant growth and water quality, it is important to evaluate and improve phosphorus simulation with fertilization in EPIC and its fate and transport in SWAT in future work.

## Supplementary Material

Supplement1

## Figures and Tables

**Table 1:** Summary of Integrated Modeling Simulations

Simulations	Models	Domain	Period	Integrated input	Evaluation focus

1. Agriculture	FEST-C EPIC	Agricultural fields in CONUS	2010, 2011, 2012	WRF/CMAQ daily weather and N deposition	Water budget and irrigation demand, yield, N budget with fertilization
2. Atmosphere	WRF/CMAQ	12-km CONUS domain	2011	FEST-C EPIC	Ambient gas-phase NH_3_ concentration, NH_4_^+^ wet deposition
3. Hydrology and Water Quality	SWAT	Eight-digit HUC watersheds in the Mississippi River Basin (MRB)	2010, 2011, 2012	FEST-C EPIC, WRF/CMAQ daily weather and N deposition	Stream flow and dissolved N near the basin outlet to the Gulf of Mexico

Abbreviations: CMAQ: Community Multiscale Air Quality; CONUS: conterminous United States; EPIC: Environmental Policy Integrated Climate; FEST-C: Fertilizer Emission Scenario Tool for CMAQ; SWAT: Soil and Water Assessment Tool; WRF: Weather Research and Forecast.

## Key Points:

- Modeling components representing agriculture, atmosphere, and hydrology are integrated and evaluated
- Integrated agriculture, hydrology, and water quality respond to different-year weather conditions as expected
- Air quality linked with simulated agriculture improves NH_3_ flux estimation and results in better performance of N cycling in atmosphere
