# Supplementary material for: An Integrated Agriculture, Atmosphere, and Hydrology Modeling System for Ecosystem Assessments
Source: J Adv Model Earth Syst. Author manuscript; Available in PMC 2021 Jun 11. (PMC8193828; doi:10.1029/2019MS001708)
Supplement: Supplement1 [file NIHMS1554672-supplement-Supplement1.docx]

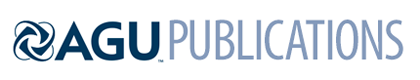


*Journal of Advances in Modeling Earth Systems*

Supporting Information for

**An integrated agriculture, atmosphere, and hydrology modeling system for ecosystem assessments**

L. Ran^1^, Y. Yuan^1^, E. Cooter^2^, V. Benson^3^, D. Yang^4^, J. Pleim^1^, R. Wang^5^, and J. Williams^6^

^1^United States Environmental Protection Agency, Research Triangle Park, North Carolina, USA

^2^United States Environmental Protection Agency, Research Triangle Park, North Carolina, USA (retired)

^3^Benson Consulting, Columbia, Missouri, USA

^4^University of North Carolina at Chapel Hill, Chapel Hill, North Carolina, USA

^5^Department of Land, Air, and Water Resources, University of California, Davis, CA, USA

^6^Blackland Research & Extension Center, Texas A&M University, Texas, USA

Corresponding author: Limei Ran (LimeiRan12@gmail.com)

**Contents of this file**

**Text S1.** FEST-C agricultural land from NLCD/MODIS pasture and cropland.

**Text S2.** FEST-C NLCD agricultural land compared with USDA NASS COA areas.

**Text S3.** Agricultural production type changes over the three NLCD years.

**Text S4.** Average yearly dry and wet N deposition changes over the two 5-year average periods.

**Figure S1**. Corn grain (types 11 and 12, top row plots) and soybean (types 31 and 32, bottom row plots) production area percent in CMAQ 12 km modeling grid cells from 2001 (left column plots), 2006 (middle column plots), and 2011 (right column plots) FEST-C land use data sets.

**Figure S2.** Two 5-year average (2002 to 2006 and 2006 to 2010) WRF/CMAQ dry and wet N deposition (kg ha^-1^ yr^-1^, left side of the bar chart) in oxidized (DryN_OX, WetN_OX), reduced (DryN_RE, WetN_RE), and organic (WetN_OR) forms and average precipitation (mm yr^-1^, right side of the bar chart) summarized as average over CONUS (top plot) and US agricultural land (bottom plot).

**Table S1.** 42 rainfed and irrigated agricultural production types simulated in FEST-C EPIC.

**Table S2.** Total agricultural land from FEST-C NLCD (A) and USDA Census of Agriculture (B) and their differences (C) over the conterminous US.

**Introduction**

The supplementary materials provide additional descriptions, figures, and tables to help describe the production types and agricultural land used in FEST-C modeling and its comparison with USDA COA areas and to explain the trend of the two N deposition data sets available to EPIC simulations.

Text S1. FEST-C agricultural land from NLCD/MODIS pasture and cropland.

NLCD Pasture/Hay (class 81) areas are used to compute area fractions of managed grassland types and NLCD Cultivated Crops (class 82) areas are for cropland types in US. Because Mexico has limited census information accessible by the public, the current SA for FEST-C only generates the 42 production type fractions for domain grid cells in US and Canada as displayed in Figure 2. It is obvious that most of managed grassland is concentrated in the humid east of US. The discontinuity of the pasture coverage along the US and Canada border in the northwest is caused by classification and accuracy discrepancies between NLCD and MODIS land cover data. While the agricultural land in Canada is concentrated in the western central from MODIS data, the majority of it in US is located in the Mississippi River Basin. Note that the FEST-C EPIC simulation is configured for the US only in the current release.

Text S2. FEST-C NLCD agricultural land compared with USDA NASS COA areas.

Following the approach of partitioning the USDA NASS COA data into the possible equivalent of NLCD managed grassland and cropland categories by Maxwell et al. (2008) and Goslee (2011), Table S2 shows the FEST-C NLCD (A, aggregated from FEST-C 42 types at 12 km grid cells) and USDA COA (B, aggregated from state-level COA reports) agricultural land and their differences (C) over CONUS. The total managed grassland and cropland from COA decrease slightly from 2002 to 2012 (Table S2. B). The cropland areas from NLCD and COA show very good agreement overall for the similar period, though the COA reporting is one year behind NLCD. The NLCD managed grassland is much smaller than the COA grassland because of the inclusion of rangeland in COA. But, the difference of managed grassland is likely to be much smaller than the amount (around 3 times) displayed in the table. Irrigated land is underestimated by around 26% in the FEST-C agriculture land for 2001 and 2006 due to limited irrigation reporting by production types at the county level.

Text S3. Agricultural production type changes over the three NLCD years.

For example, there is much more hay production land along with reduced other grass land since 2006. The corn grain area has a significant increase since 2006 due to the high demand for corn-based ethanol production with most of the increase concentrated in the Corn Belt region (Figures S1a, S1b, S1c). In contrast, the soybean production land in 2006 shows a decrease in the same region (Figures S1d, S1e, S1f) which is likely caused by the production conversion to corn grain for meeting the biofuel demand. In 2011 soybean production land recovers to a level similar to 2001 (Figure 3). The area for rainfed Other Crop shows a decreasing trend over the period but the area for irrigated other crops shows a big jump in 2011 which is caused by better irrigation reporting in the 2012 COA as indicated in Table S2.

Text S4. Average yearly dry and wet N deposition changes over the two 5-year average periods.

Figure S2 displays the average yearly dry and wet N deposition in the oxidized, reduced, and organic forms (left side of the bar chart) and average precipitation (right site of the bar chart) over the two periods for CONUS excluding ocean areas (a) and agricultural lands (b). With the control of NOx emissions, oxidized-N deposition decreased by about 19% for both the CONUS and agricultural land areas over the two periods. However, the reduced-N dry deposition increased while the reduced-N wet deposition decreases associated with slightly lower precipitation in the later period.


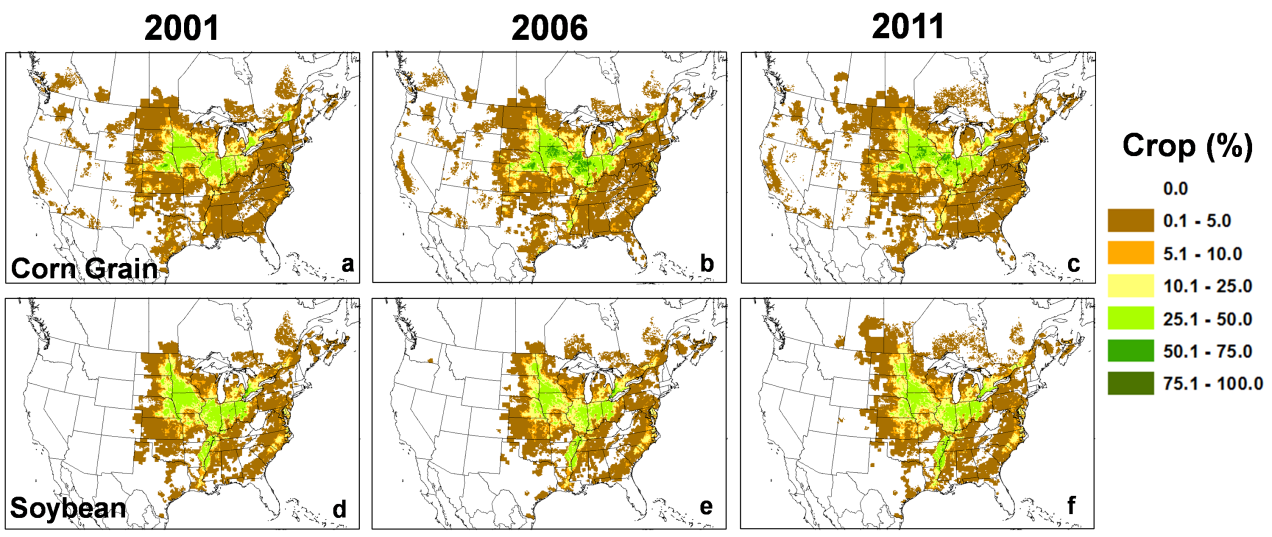


Figure S1. Corn grain (types 11 and 12, a, b, c) and soybean (types 31 and 32, d, e, f) production area percent in CMAQ 12 km modeling grid cells from 2001 (a, d), 2006 (b, e), and 2011 (e, f) FEST-C land use data sets.


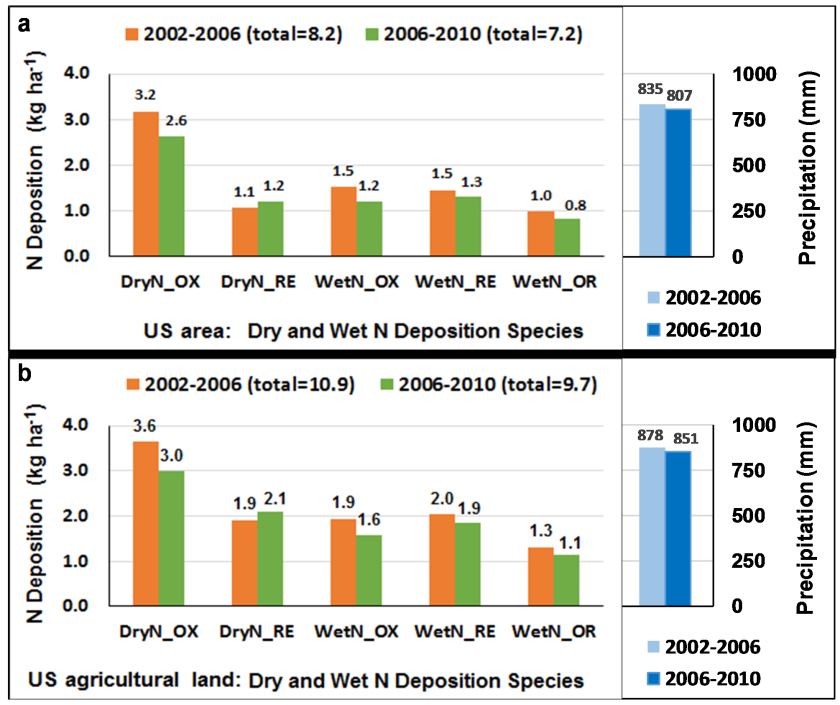


Figure S2. Two 5-year average (2002 to 2006 and 2006 to 2010) WRF/CMAQ dry and wet N deposition (kg ha^-1^ yr^-1^, left side of the bar chart) in oxidized (DryN_OX, WetN_OX), reduced (DryN_RE, WetN_RE), and organic (WetN_OR) forms and average precipitation (mm yr^-1^, right side of the bar chart) summarized as average over CONUS (a) and US agricultural land (b).

| **Type#** | **Type Name** | **Type#** | **Type Name** | **Type#** | **Type Name** |
| --- | --- | --- | --- | --- | --- |
| 1 | Hay | 15 | Cotton | 29 | SorghumSilage |
| 2 | Hay_ir | 16 | Cotton_ir | 30 | SorghumSilage_ir |
| 3 | Alfalfa | 17 | Oats | 31 | Soybeans |
| 4 | Alfalfa_ir | 18 | Oats_ir | 32 | Soybeans_ir |
| 5 | Other_Grass | 19 | Peanuts | 33 | Wheat_Spring |
| 6 | Other_Grass_ir | 20 | Peanuts_ir | 34 | Wheat_Spring_ir |
| 7 | Barley | 21 | Potatoes | 35 | Wheat_Winter |
| 8 | Barley_ir | 22 | Potatoes_ir | 36 | Wheat_Winter_ir |
| 9 | BeansEdible | 23 | Rice | 37 | Other_Crop |
| 10 | BeansEdible_ir | 24 | Rice_ir | 38 | Other_Crop_ir |
| 11 | CornGrain | 25 | Rye | 39 | Canola |
| 12 | CornGrain_ir | 26 | Rye_ir | 40 | Canola_ir |
| 13 | CornSilage | 27 | SorghumGrain | 41 | Beans |
| 14 | CornSilage_ir | 28 | SorghumGrain_ir | 42 | Beans_ir |

Table S1. 42 rainfed and irrigated agricultural production types simulated in FEST-C EPIC.

Table S2. Total agricultural land from FEST-C NLCD (A) and USDA Census of Agriculture (B) and their differences (C) over the conterminous US.
